# Using micro-computed tomography to reveal the anatomy of adult *Diaphorina citri* Kuwayama (Insecta: Hemiptera, Liviidae) and how it pierces and feeds within a citrus leaf

**DOI:** 10.1038/s41598-020-80404-z

**Published:** 2021-01-14

**Authors:** Javier Alba-Tercedor, Wayne B. Hunter, Ignacio Alba-Alejandre

**Affiliations:** 1 Department of Zoology, Faculty of Sciences, University of Granada, Campus de Fuentenueva, Granada, Spain; 2 U.S. Dept. Agriculture, Agricultural Research Service, Fort Pierce, FL USA

**Keywords:** Zoology, Entomology

## Abstract

The Asian citrus psyllid (ACP), *Diaphorina citri*, is a harmful pest of citrus trees that transmits *Candidatus* Liberibacter spp. which causes Huanglongbing (HLB) (citrus greening disease); this is considered to be the most serious bacterial disease of citrus plants. Here we detail an anatomical study of the external and internal anatomy (excluding the reproductive system) using micro-computed tomography (micro-CT). This is the first complete 3D micro-CT reconstruction of the anatomy of a psylloid insect and includes a 3D reconstruction of an adult feeding on a citrus leaf that can be used on mobile devices. Detailed rendered images and videos support first descriptions of coxal and scapus antennal glands and sexual differences in the internal anatomy (hindgut rectum, mesothoracic ganglion and brain). This represents a significant advance in our knowledge of ACP anatomy, and of psyllids in general. Together the images, videos and 3D model constitute a unique anatomical atlas and are useful tools for future research and as teaching aids.

## Introduction

Since it was first discovered in Taiwan in 1907^1^, the Asian citrus psyllid (ACP) *Diaphorina citri* (Hemiptera: Liviidae) has become the most significant vector of *Candidatus* Liberibacter spp. (Huanglongbing [HLB] or citrus greening disease) in citrus orchards. HLB is the most serious disease threatening the citrus industry^2^. To date, HLB has spread to over 40 countries in Asia, Oceania and both North and South America^3, 4^.

After Witlaczil's pioneering work at the end of the nineteenth century^5, 6^ a number of studies have described various aspects of the anatomy of different psyllid species^7–56^. The majority of these descriptions and illustrations are based on microscopy techniques that require insect dissection to enhance visibility of internal structures and organs. While dissection techniques are very useful, they have limitations because they distort the spatial arrangement of internal structures. A relatively recent technique, known as micro-computed tomography (micro-CT), which is based on X-rays, allows visualization of the internal anatomy in situ, without the need to dissect the insect; results have been validated by comparing them with classical destructive methodologies^58, 59^. Thus, micro-CT has now been used to elucidate insect anatomy; for ACP, a preliminary publication on the opportunities that micro-CT offers^60^, and two detailed studies of the male^8^ and female^7^ reproductive system have already been published, including a revision of the reproductive system of psyllids in general.

Thus, the main aim of the current study was to fill any knowledge gaps by extending anatomical studies of ACP to the entire anatomy (external and internal), and configure a 3D anatomical atlas. Here we present an extensive application of the micro-CT techniques to reveal, in detail, the entire anatomy (excluding the reproductive systems and external abdominal terminalia of both sexes that we have already published^7, 8^) and a 3D reconstruction of an adult feeding on a leaf.

We also present videos as Supplementary information; these provide an accurate view of the actual position and internal components of the organs and structures. We present spinning animations, using different rotation axes, that permit detailed observation of the structures from different 3D perspectives (Supplementary Videos S1–S11), and a 3D model that can be used on mobile devices (Supplementary 3D model S12).

Together, this increases current knowledge and helps us understand the morphology and functional anatomy of structures in their natural anatomical position, avoiding deformations that typically occur using standard dissection and/or slide preparation techniques. The high-quality rendered images and the Supplementary information (videos and 3D model) represent a unique anatomical atlas of ACP and are useful tools for future research and as teaching aids.

## Results

### External anatomy

Figures 1, 2, 3, 4, 5, 6, 7, 8 and 9a, Supplementary Videos S1–S4 and Supplementary 3D model S12.

**Figure 1:**
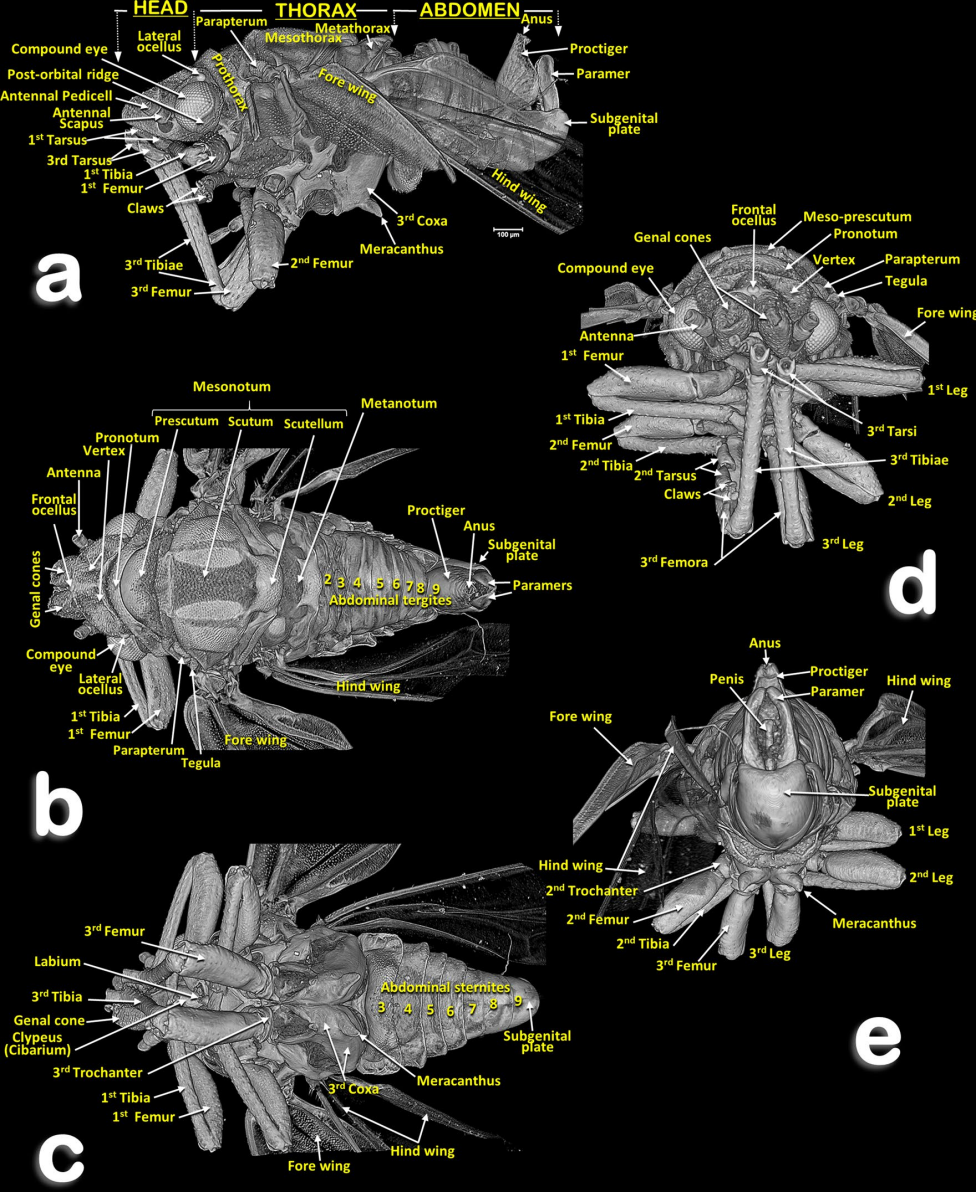
Volume-rendered images of the general external anatomy of a male *Diaphorina citri* in different views. Left-lateral (**a**), dorsal (**b**), ventral (**c**), frontal (**d**) and posterior (**e**).

**Figure 2:**
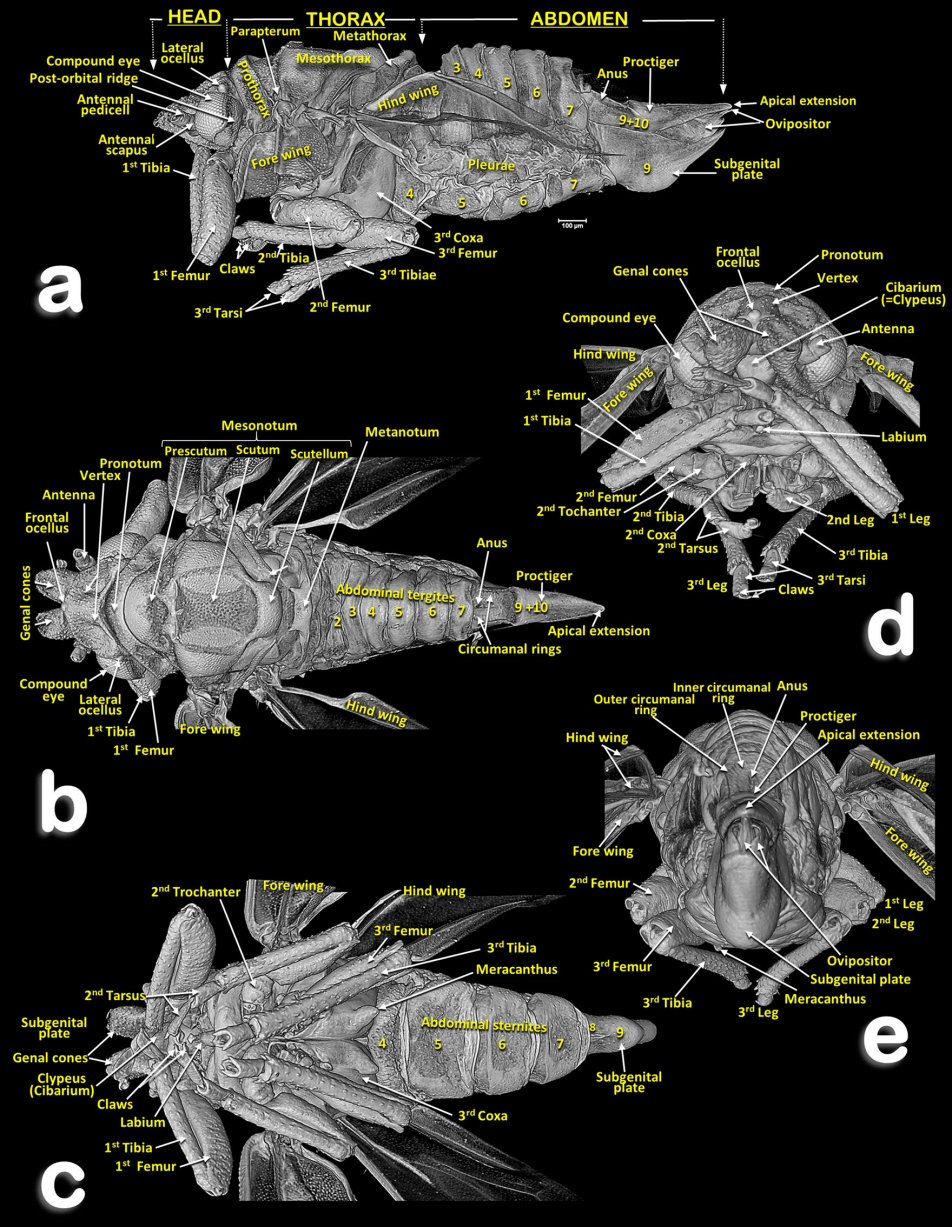
Volume-rendered images of the general external anatomy of a female *Diaphorina citri* in different views. Left-lateral (**a**), dorsal (**b**), ventral (**c**), frontal (**d**) and posterior (**e**).

**Figure 3:**
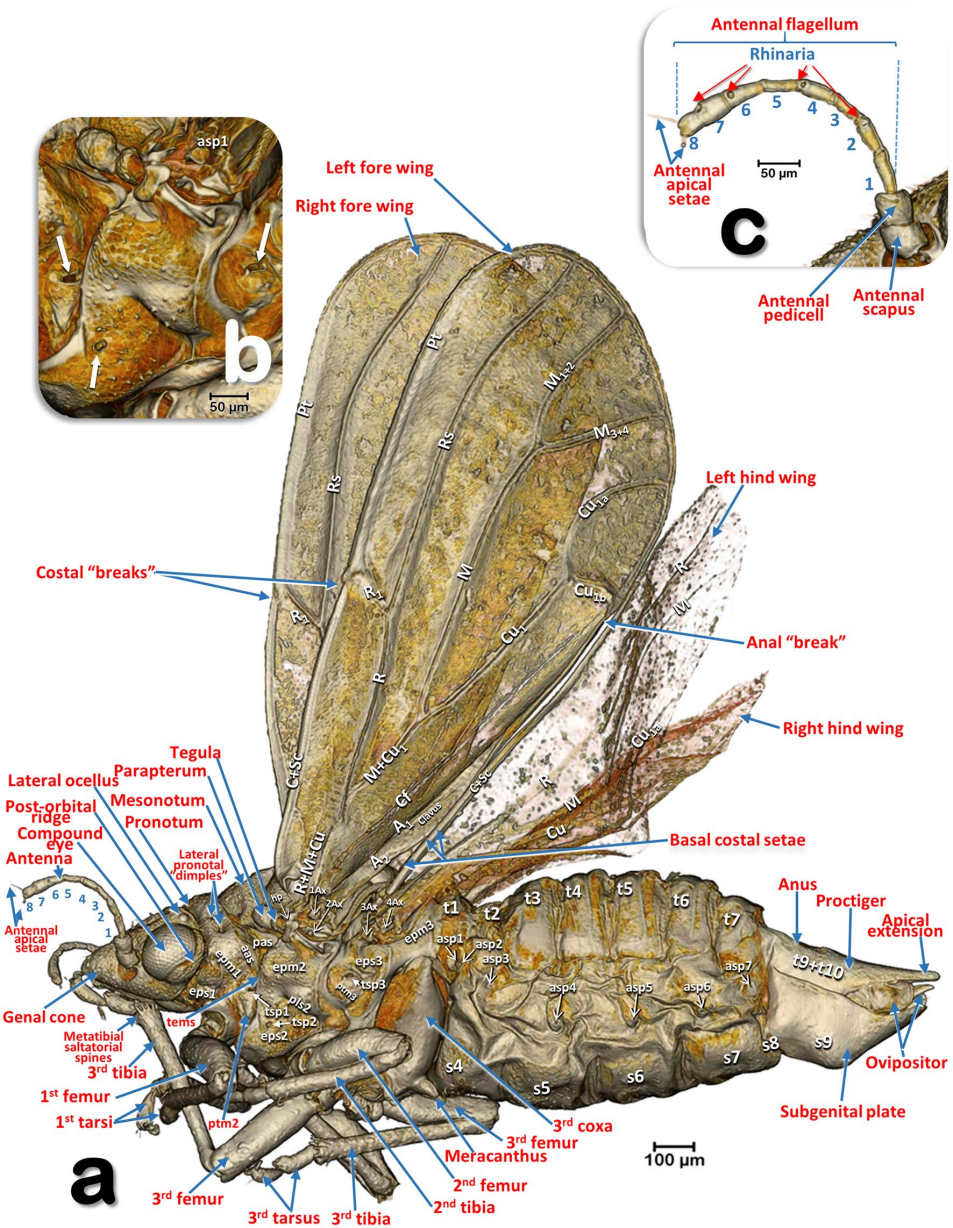
Volume-rendered images of an external left-lateral detailed view of a female *Diaphorina citri* (**a**) and a close-up detail of the pleural region of the thorax (**b**) (arrows indicate the spiracular openings), and the antenna (**c**). Abbreviations: *A* = anal vein; *aas* = anterior accessory sclerite; *asp* = abdominal spiracle; *Ax* =Axillary sclerite; *C* = costa vein; *Cf* = cubital fold; *Cu* = cubital vein; *epm* = epimeron; *eps* = episternum; *hp* = humeral plate; *M* = median vein; *pas* = posterior accessory sclerite; *pls* = pleural sulcus; *Pt* = pterostigma; *ptm2* = mesothoracic peritreme; *ptm3* = metathoracic peritreme; *R* = radius vein; *Rs* = radial sector vein; *s* = abdominal sternite; *t* = abdominal tergite; *tems* = transepimeral sulcus; *tsp1* = prothoracic spiracle; *tsp2* = mesothoracic spiracle; *tsp3* = metathoracic spiracle. Abdominal tergites and sternites are labelled sequentially with the letter ‘t’ and ‘s’, respectively.

**Figure 4:**
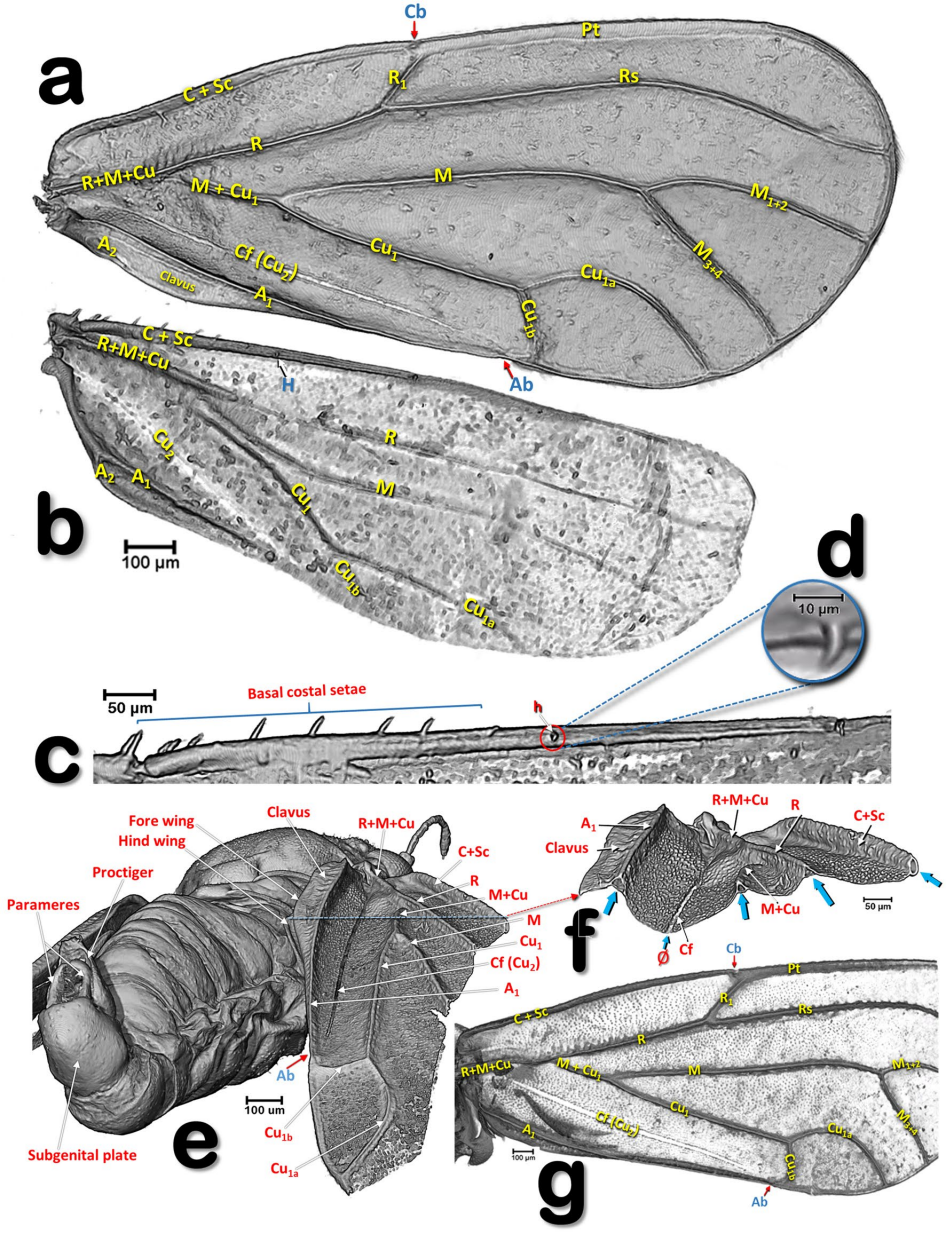
Volume-rendered images of the wings and veins of *Diaphorina citri*: left wings of a female in a ventral view (**a**–**d**) and right forewing of a male (**g**), in different views showing the dorsal surface (**e**–**g**). General shape of the wings and veins (**a**, **b**). Details of the dorsal fore basal margin of the hind wing (**c**). Detail of the hamulus (**d**). Right-lateral posterior view of a male showing veins and folds of the right wing (**e**). Details of the basal right forewing (**f**), as a result of a virtual cut marked with a red dashed line in e (blue arrows indicate the transversal cut of the vein tubes, the symbol ‘Ø’ indicates the non-tubular structure of the cubital fold. Abbreviations: *A* = anal vein; *Ab* = anal break; *C* = costa vein; *Cb* = costal break; *Cf* = cubital fold; *Cu* = cubital vein; *H* = hamulus; *M* = median vein; *R* = radius vein; *Rs* = radial sector vein.

**Figure 5:**
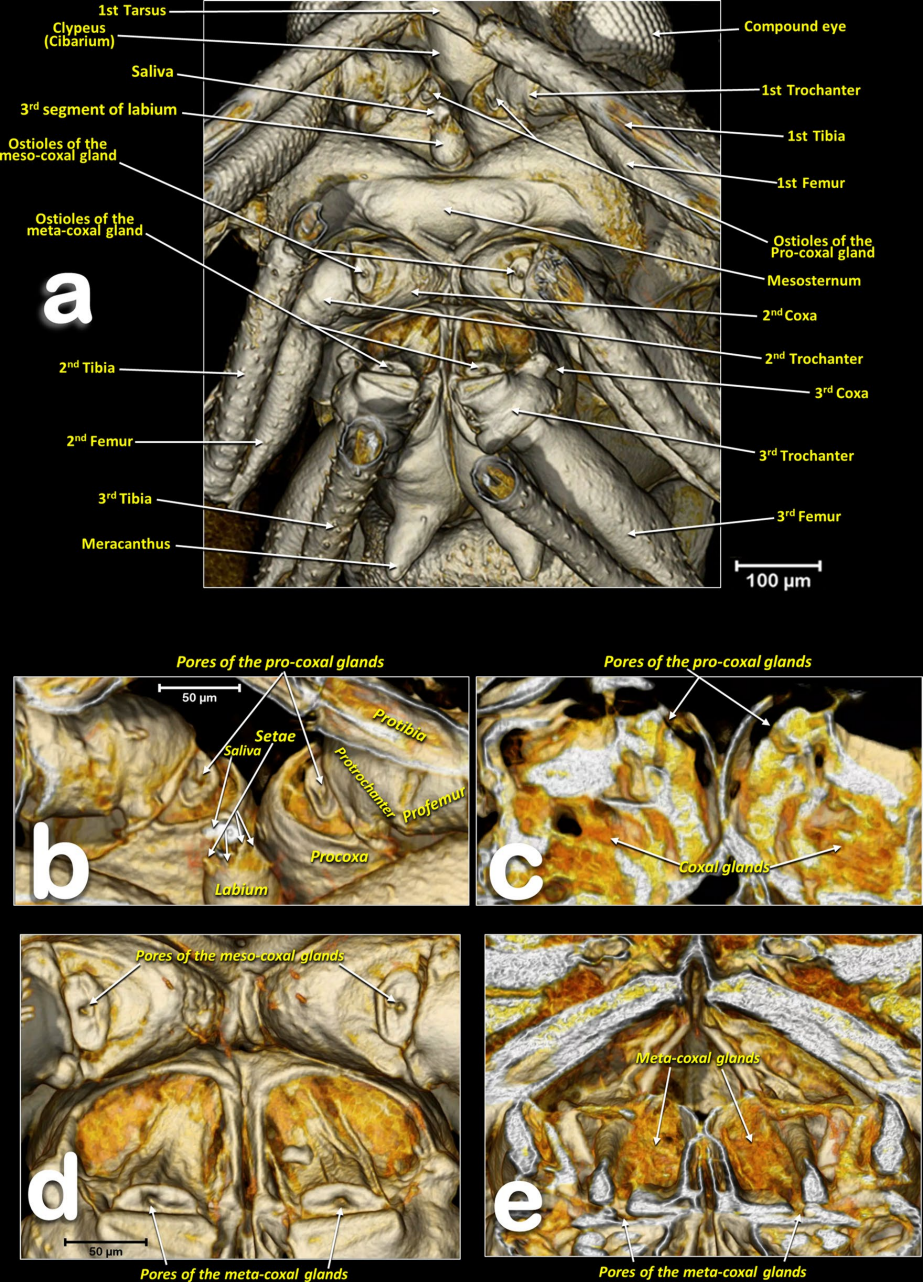
Volume-rendered images of the anterior half of a female *Diaphorina citri*, in a ventral view, showing the main anatomical structures and the ostioles of the coxal glands (**a**). Close-up the ostioles of the coxal glands (**b**–**d**), and antero-posterior oblique virtual cuts showing the coxal gland tissue inside the coxae (**c**–**e**).

**Figure 6:**
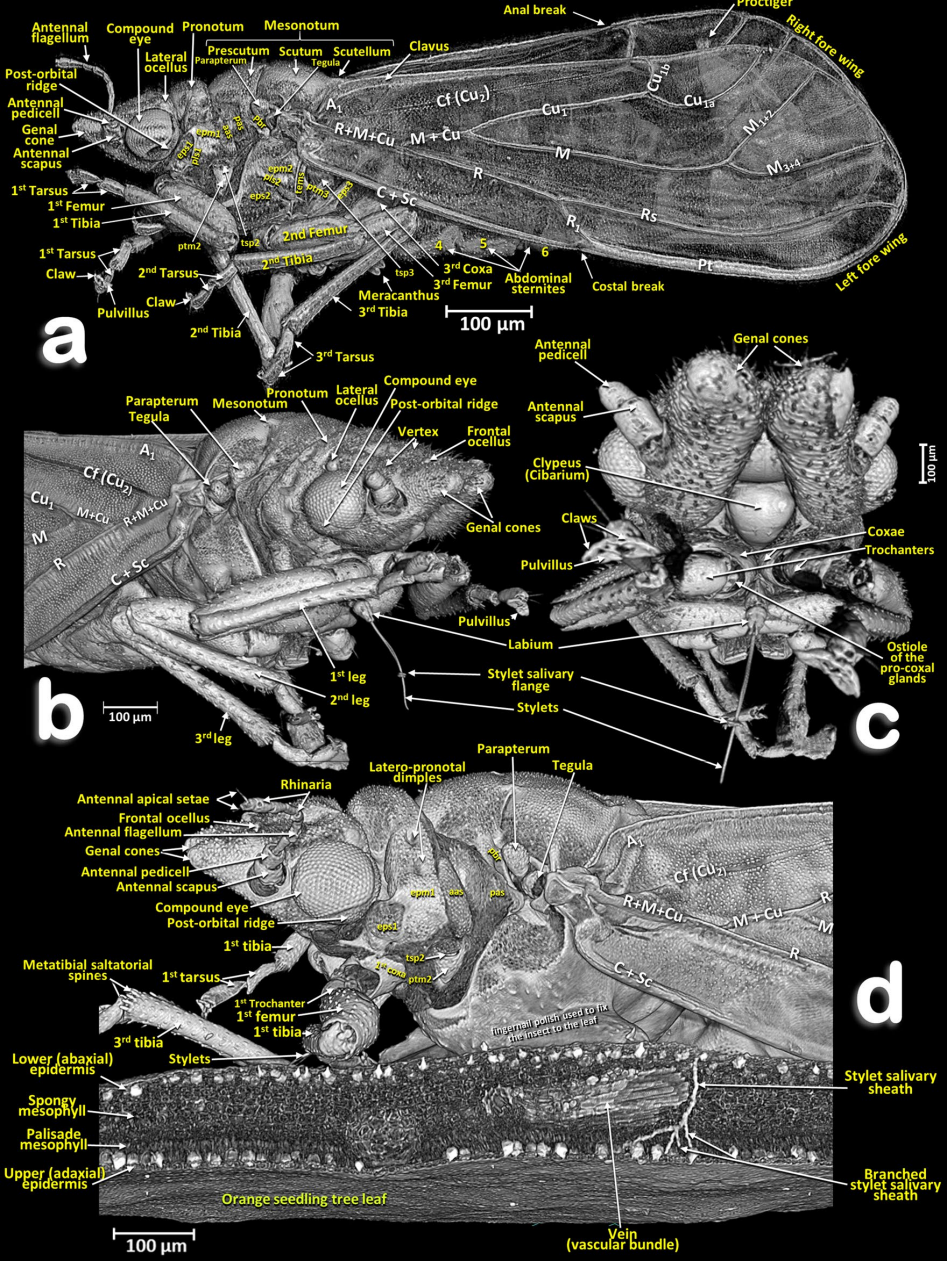
Volume-rendered images of a male *Diaphorina citri* in different perspective views. Left-lateral (**a**), right-frontal (**b**), ventro-frontal (**c**), and a left-fore half of an individual feeding on an orange seedling tree leaf (**d**). Abbreviations: *A* = anal vein; *aas* = anterior accessory sclerite; *C* = costa vein; *Cb* = costal break; *Cf* = cubital fold; *Cu* = cubital vein; *epm* = epimeron; *eps* = episternum; *M* = median vein; *pas* = posterior accessory sclerite; *pbr* = prealar bridge; *pls* = pleural sulcus; *Pt* = pterostigma; *ptm2* = mesothoracic peritreme; *ptm3* = metathoracic peritreme; *R* = radius vein; *Rs* = radial sector vein; *tems* = transepimeral sulcus; *tsp2* = mesothoracic spiracle; *tsp3* = metathoracic spiracle.

**Figure 7:**
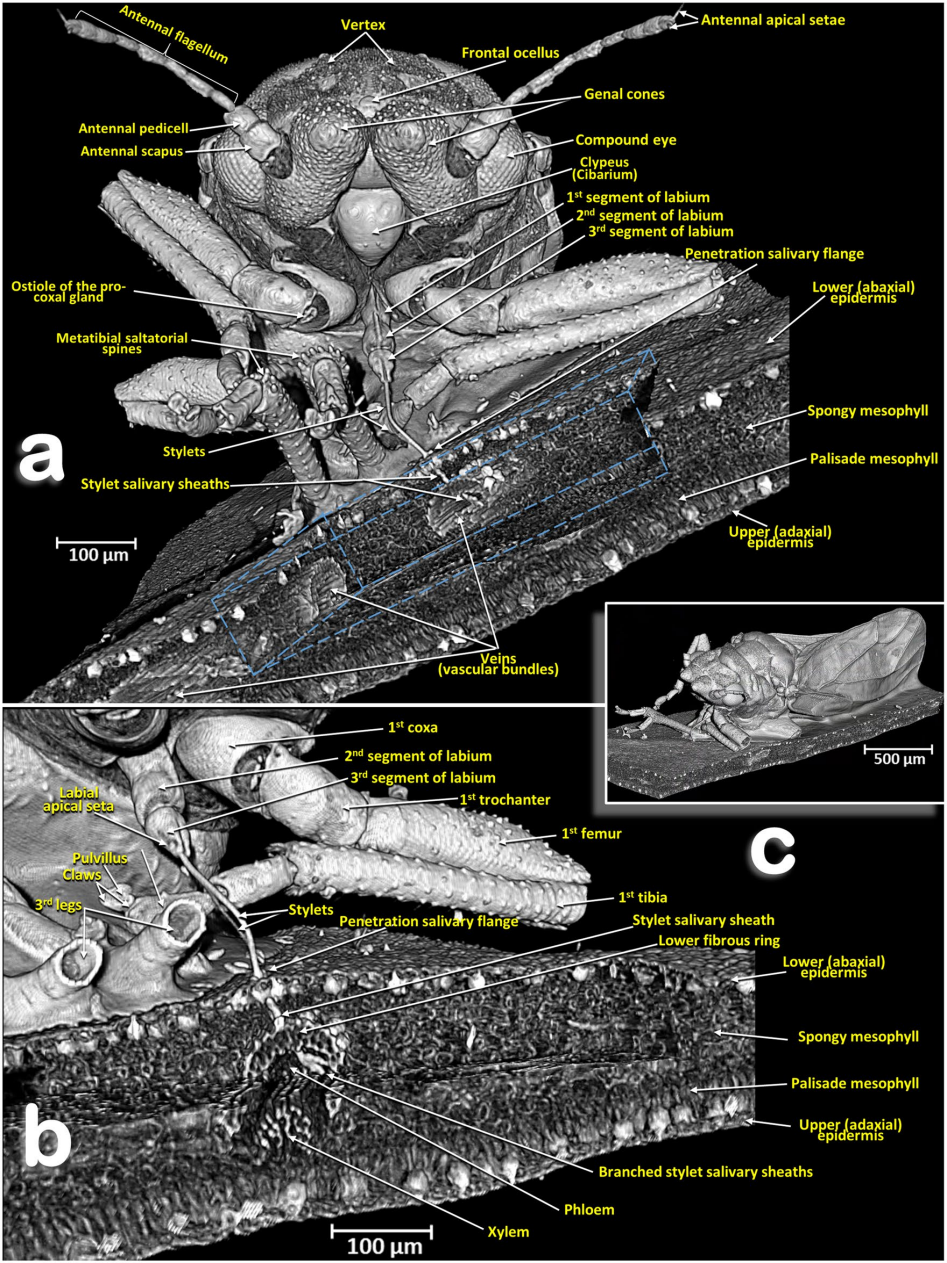
Volume-rendered close-up images (**a**, **b**) of a male *Diaphorina citri* feeding on an orange seedling tree leaf (**c**) in two perspective views: frontal (**a**) right-frontal (**b**). In (**a**) the virtual box-cut made to visualize the stylets and salivary sheaths inside the leaf and vascular bundle is indicated with a dotted blue line.

**Figure 8:**
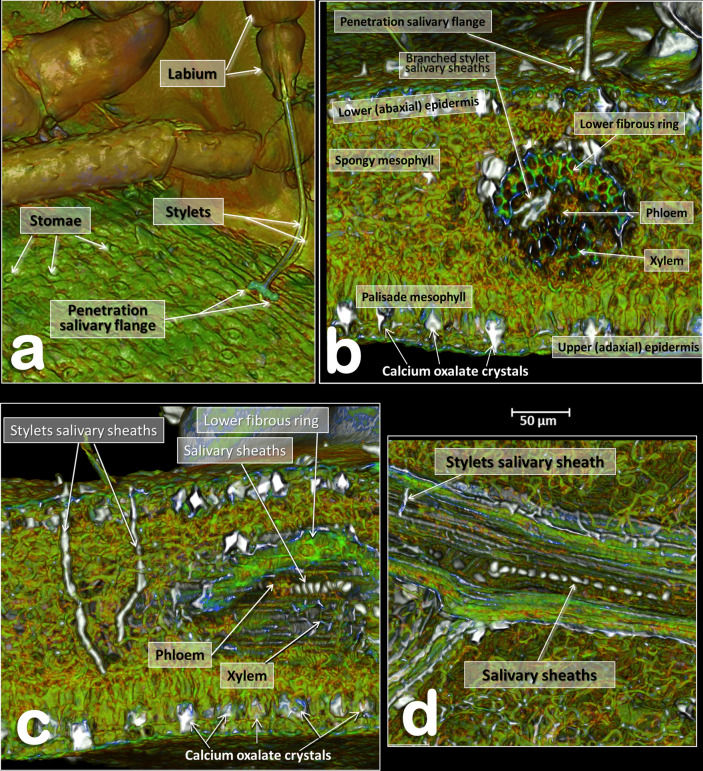
Volume-rendered images of details of an orange seedling tree leaf where a male *Diaphorina citri* was feeding on the abaxial surface of the leaf. Detail of the stylets and penetration point (**a**). Transverse cut of the leaf showing the penetration point of the stylets, different anatomical structures of the leaf and the tip of the stylets as branched stylet salivary sheaths inside the phloem (**b**). Transverse cut of the leaf showing abandoned stylets and salivary sheaths inside the phloem of a vein (**c**). Middle plane-cut, between the lower and upper surface of the leaf, at the level of the vein are shown both in a transversal cut (**c**) and in a longitudinal cut (**d**).

**Figure 9:**
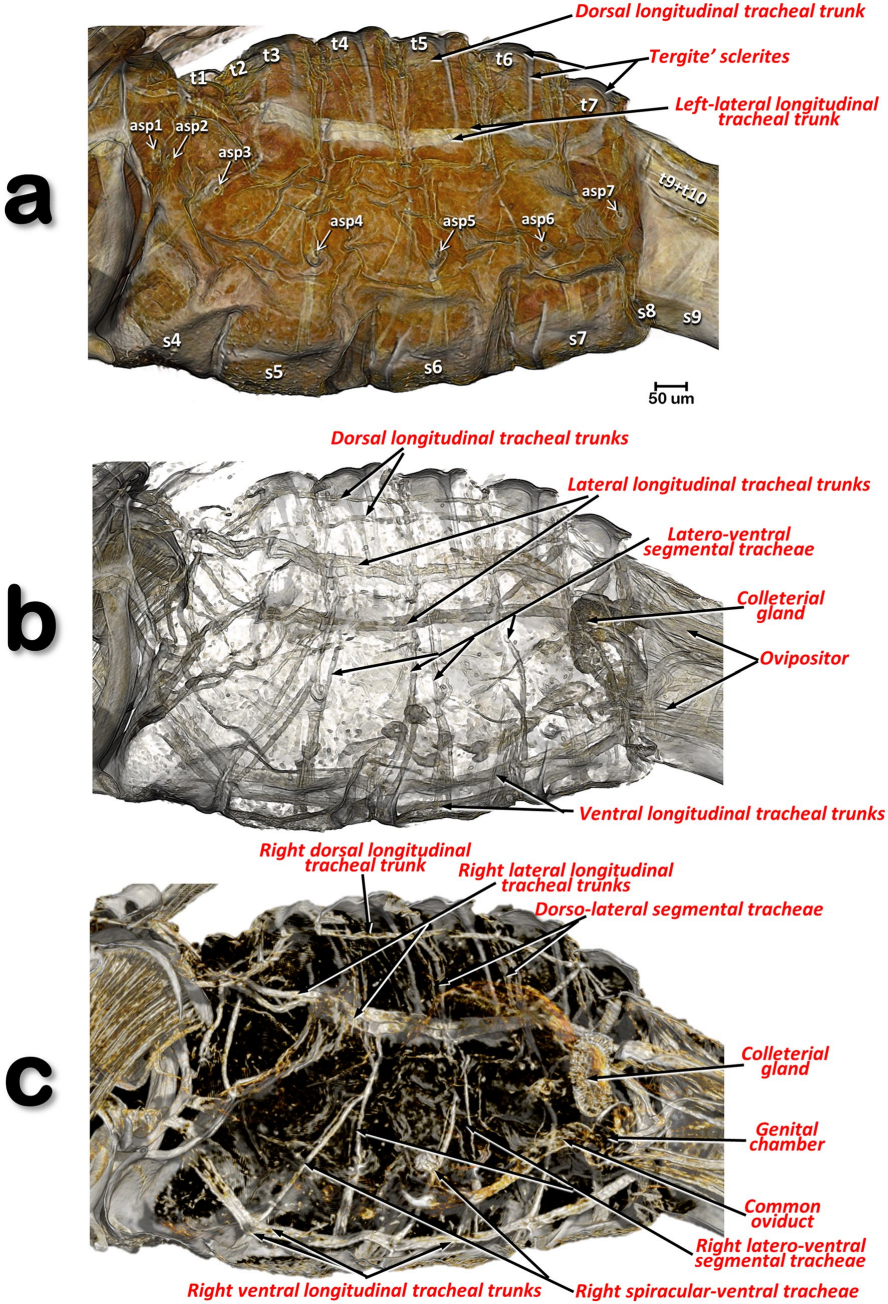
Volume-rendered images of a left-lateral view of the abdomen of a female *Diaphorina citri*, virtually cleared to show the respiratory system. Slightly cleared (**a**) and cleared (**b**, **c**), permitting observation of the tracheal tubular system. Internal view of the right lateral half (**c**). Abbreviations: *asp* = abdominal spiracle; the abdominal tergites and sternites are labelled sequentially with the letter ‘t’ and ‘s’, respectively.

### Head

Male: Figs. 1a–d, 6 and 7a; female: Fig. 3a,c and Supplementary Video S1 . The head has characteristic genal cones (conical lateral structures; each elongated, ca. twice as long as wide). There is a depression each side of the vertex (Figs. 1b and 2b); The antennae (Figs. 3b, 6 and 7a) each have a scapus, broad pedicel and a flagellum comprised of eight articles (ca. ¼ narrower than pedicel). Openings of olfactory sensilla (rhinaria) are visible in the 2nd, 4th, 6th and 7th flagellar articles, and on the 8th are two apical setae (the length of the inner one is half the length of the outer) (Fig. 3c). The compound eyes are found laterally, each with a characteristic post-orbital ridge; three ocelli (one frontal, situated dorsally, and two lateral), are situated in a dorsolateral position above the compound eyes and close to the posterior margin of the head. A conspicuously bulging clypeus (cibarium) appears ventrally (Figs. 2d, 5a, 6c and 7a), with two small rounded protuberances (Fig. 15f). The labium appears ventrally, slightly posterior and between the fore coxae (Figs. 1c, 2c, 3a,b, 6c and 7a,b). The labium has three segments: the 1st segment is the longest; the 3rd segment has a conical shape, a long anterior slit, a short posterior apical slit, and apical setae (Figs. 1c, 2c,d, 5a,b, 6b,c, 7a,b, 8a, 15, 16e and Supplementary Videos S3, S4).

### Thorax

Male: Figs. 1, 6 and 7; female: Figs. 2, 3a, 5, and Supplementary Video S1. The pronotum has two, lateral ‘dimples’ on each side. The mesothorax is well developed. The scutum and scutellum in divisions are very conspicuous in the mesonotum and the prescutum and narrower in the metathorax. Thoracic pleurae have clearly visible sclerites, including the episternal and epimeral ones, and one spiracular opening on the pleural side of each segment (Figs. 3a, 6a,d and Supplementary Video S1). The legs are arranged forwards; the hind ones are clearly longer with enlarged/longer coxae and conspicuous metatibial saltatorial spines. The tarsi have two claws and a ventral bilobed enlarged pad-like pulvillus (Fig. 6a,b). Meta-coxae are prolonged in a pointed structure, and the meracanthi (from a ventral view) appear divergent (Figs. 1c, 2c).

Axillary sclerites of the wings are clearly visible on the base of the wing attachments on the thorax wall (Fig. 3a), as well as the parapterum and tegula (Figs. 1a, 2a, 3a, 6a,b,d and Supplementary video S1).

#### Wings

Wings have reduced venation (Figs. 3a, 4a,b,g, 6a,b,d and Supplementary video S1). The costal and subcostal veins end together in the distal marginal third, forming a costal break; similarly, on the posterior margin the anal vein interrupts forming an anal break. A narrow pterostigma is present on the anterior distal margin of the forewings. On the basal posterior margin of the forewing the anal vein A1 delimits the clavus area. On the basal fore margin of the hind wings there are ca. ten stout setae (Fig. 4b,c) and a hook, the hamulus (Fig. 4d), permitting attachment to the fore wing for synchronized flight movements. The dorsal surface of the fore wings is covered with microtrichia giving a sandpaper appearance (see Fig. 4e–g).

#### Coxal glands

On the distal membrane of each coxa (between the coxa and the trochanter) is an oval sclerite, with a central hole (ostium) corresponding to the external mouth of the coxal gland (Figs. 5a,b,d and 6c).

### Abdomen

The abdominal tergites/sternites are shown in Figs. 1b,c, 2b,c and 3a. On the pleurae of abdominal segments 1–7 there are seven pairs of respiratory spiracles (Figs. 3a, 9a and Supplementary Video S1). The posterior abdominal segments (terminalia) show clear sexual differences: the male terminalia (Fig. 1a–c,e) have two parameres (the penis is located interiorly and between them, Fig. 1e) and an elevated proctiger that includes the anus (see more details of the male terminalia in our previous paper^8^); the female terminalia (Figs. 2a–c,e, 3a and Supplementary Video S1) have a pointed appearance, especially in the dorsal view. Due to an apical extension (Fig. 2b), the anus is on the base of the proctiger and it is surrounded by double concentric circum-anal rings, with pori corresponding to the external mouth of the wax glands (see more details of the female terminalia in our previous paper^7^).

### Internal anatomy

Figures 5c,e, 9, 10, 11, 12, 13, 14, 15, 16, 17, 18 and 19 and Supplementary Videos S1–S11.

**Figure 10:**
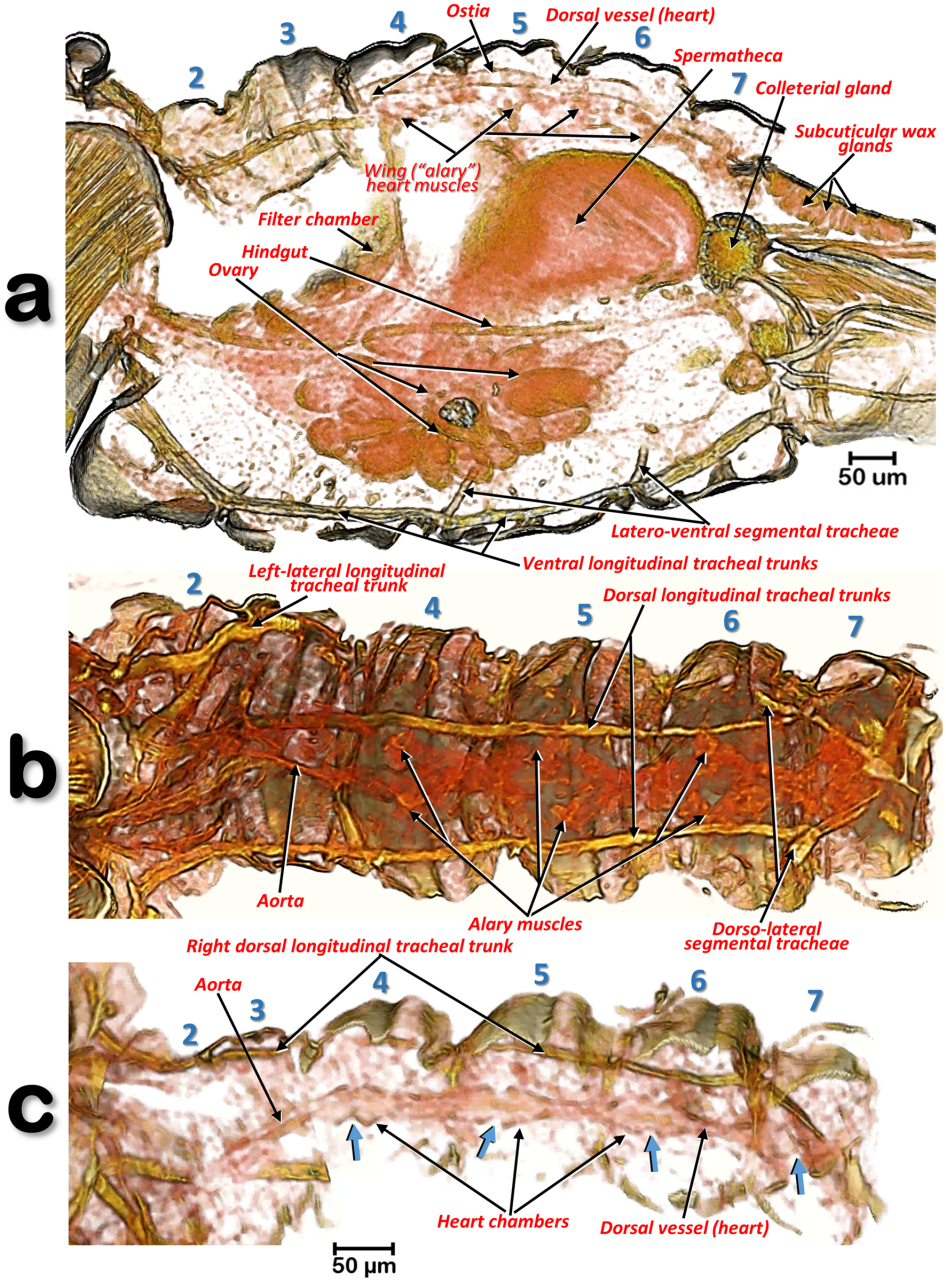
Volume-rendered images of different slice cuts of the abdomen of a female *Diaphorina citri*. Abdominal oblique sagittal slice, at the level of the dorsal vessel and left ventral tracheal trunk (**a**). Internal view of the dorsal abdominal region (**b**). External view of the dorsal vessel (tergites have been virtually removed by software) (**c**). In (**c**) the blue arrows indicate the ostia of the heart chambers. Abdominal segments are numbered sequentially.

**Figure 11:**
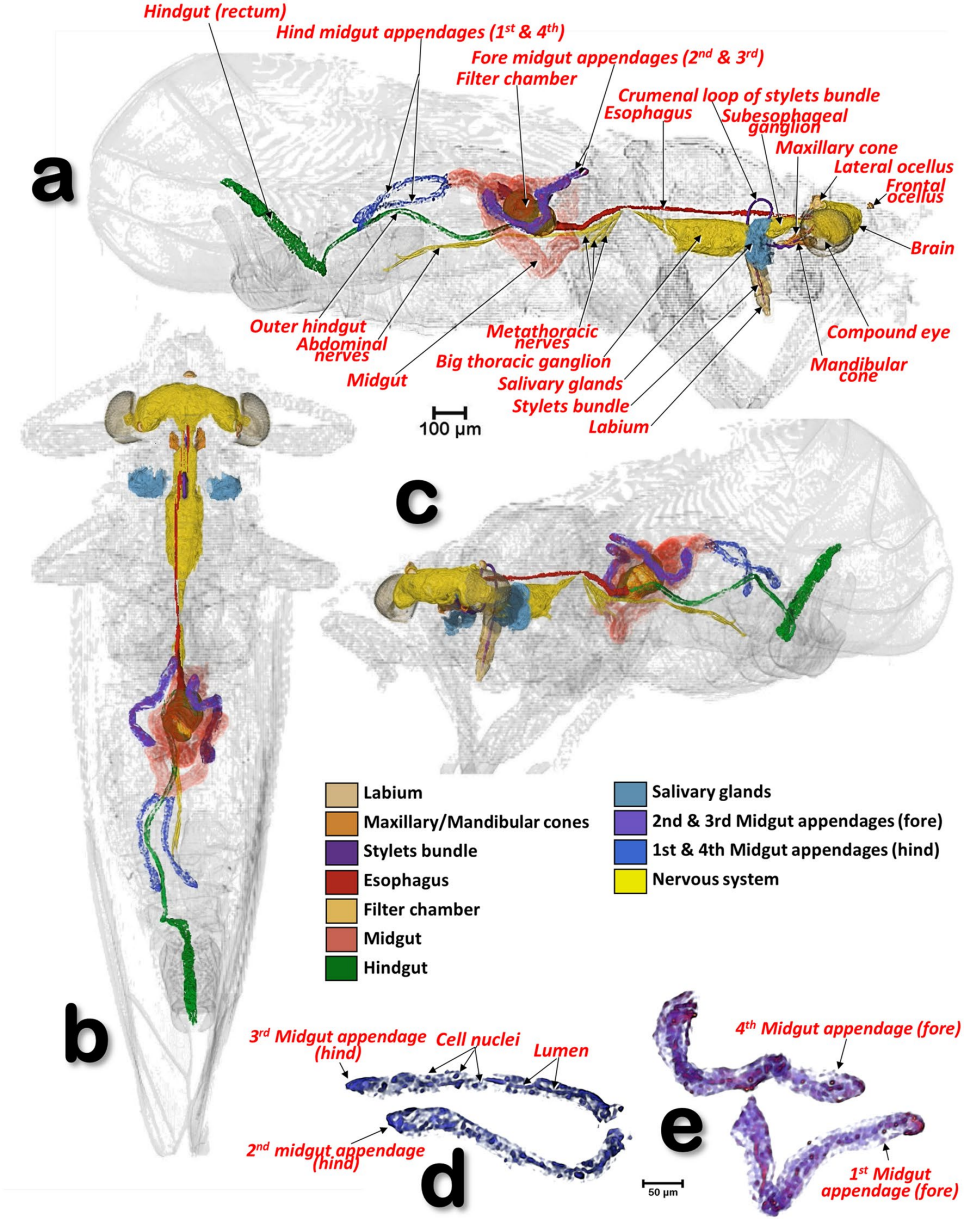
Volume-rendered images of a male *Diaphorina citri* showing the general position of the nervous system, and the digestive system (including the salivary glands), in different perspective views. Right-lateral (**a**), dorsal (**b**), and left-frontal (**c**). Details of the midgut appendages (**d**, **e**).

**Figure 12:**
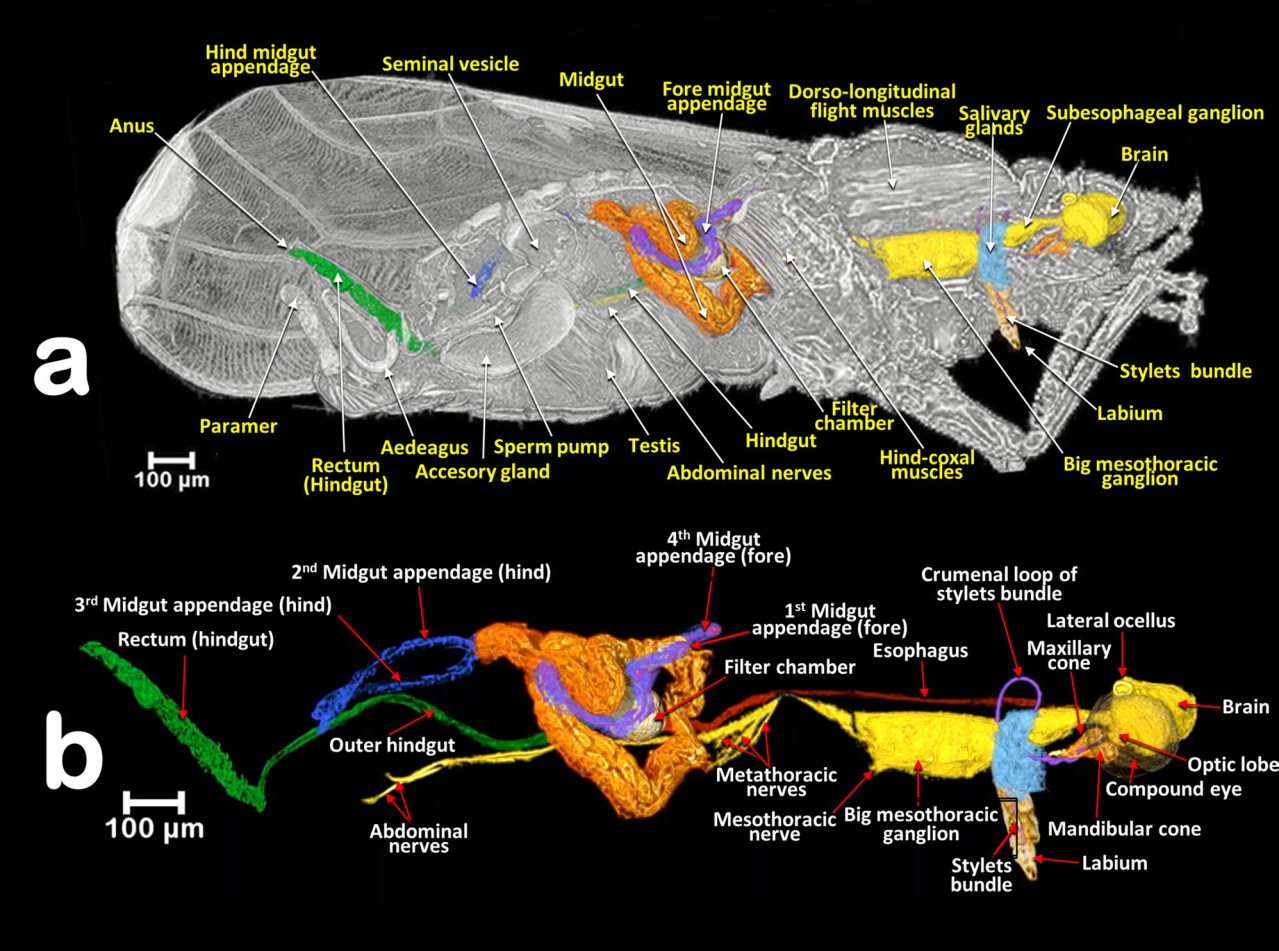
Volume-rendered images of a male *Diaphorina citri* in a right-lateral view, showing the feeding apparatus, the digestive system (including the salivary glands) and nervous system. Medial sagittal view (**a**). Isolated segmented organs and structures (**b**).

**Figure 13:**
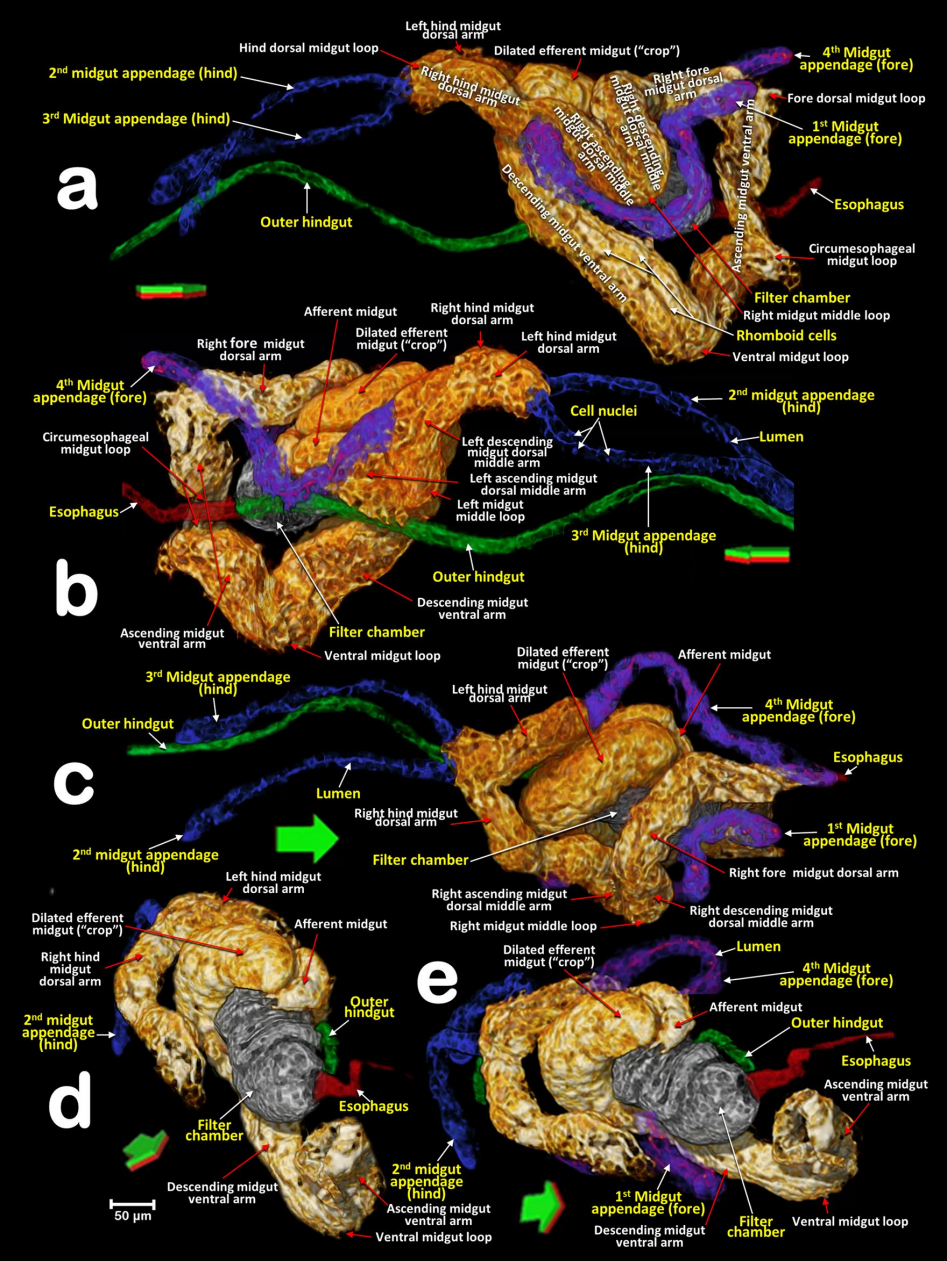
Volume-rendered close-up images of the digestive system of a male *Diaphorina citri* in different perspectives. Right-lateral (**a**), left-lateral (**b**), dorsal (**c**), right-frontal (**d**) and right-oblique-dorsal (**e**). Arrows indicate the anatomical positions. Green/red surfaces correspond with dorsal/ventral positions.

**Figure 14:**
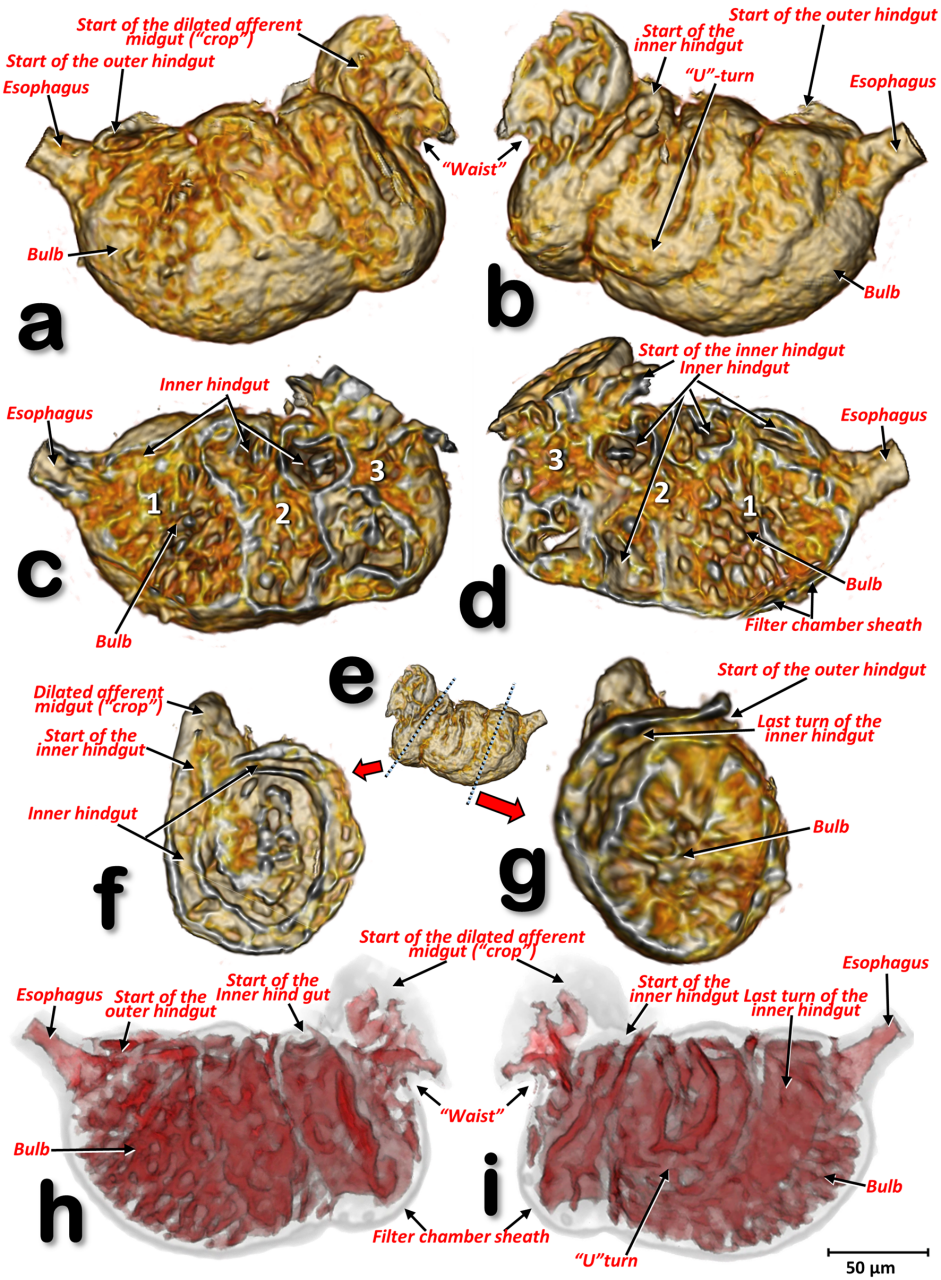
Volume-rendered close-up images of the filter chamber of a male *Diaphorina citri*, in different perspective views: left-lateral (**a**, **c**, **h**), right-lateral (**b**, **e**, **i**). Internal structure views after virtual cuts: sagittal (**c**, **d**), transverse (**f**, **g**) through positions marked with blue dotted lines in (**e**), and reconstruction of the cavities (spaces) inside the filter chamber (**h**, **i**).

**Figure 15:**
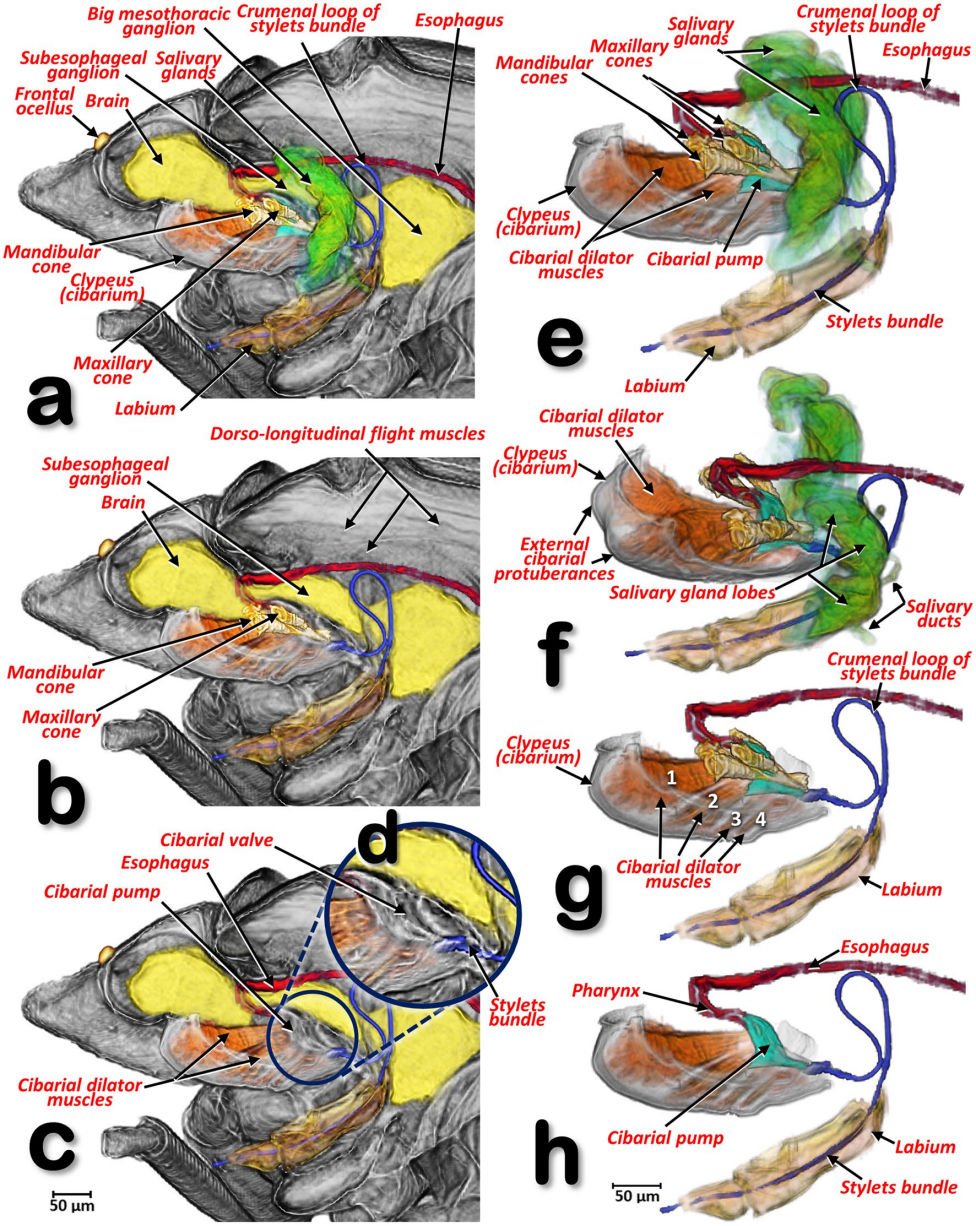
Volume-rendered images, in a left-lateral view, of the fore region of a female *Diaphorina citri*. In the left-hand column (**a**–**d**) the nervous system, the feeding apparatus, the anterior part of digestive (including the salivary glands) and cibarium are shown. The salivary glands were virtually removed using software in (**a**–**c**), and the mandibular and maxillary cones were virtually removed in (**c**). Close-up detail of the cibarial pump (**d**). In the right-hand column, the segmented structures are shown separately from the rest of the animal (**e**–**h**) (**a**, **g**, **h** = left-lateral; **f** = left-lateral, slightly dorsal-oblique). The salivary glands were virtually removed using software from (**f**–**h**), and the mandibular and maxillary cones were virtually removed from (**h**).

**Figure 16:**
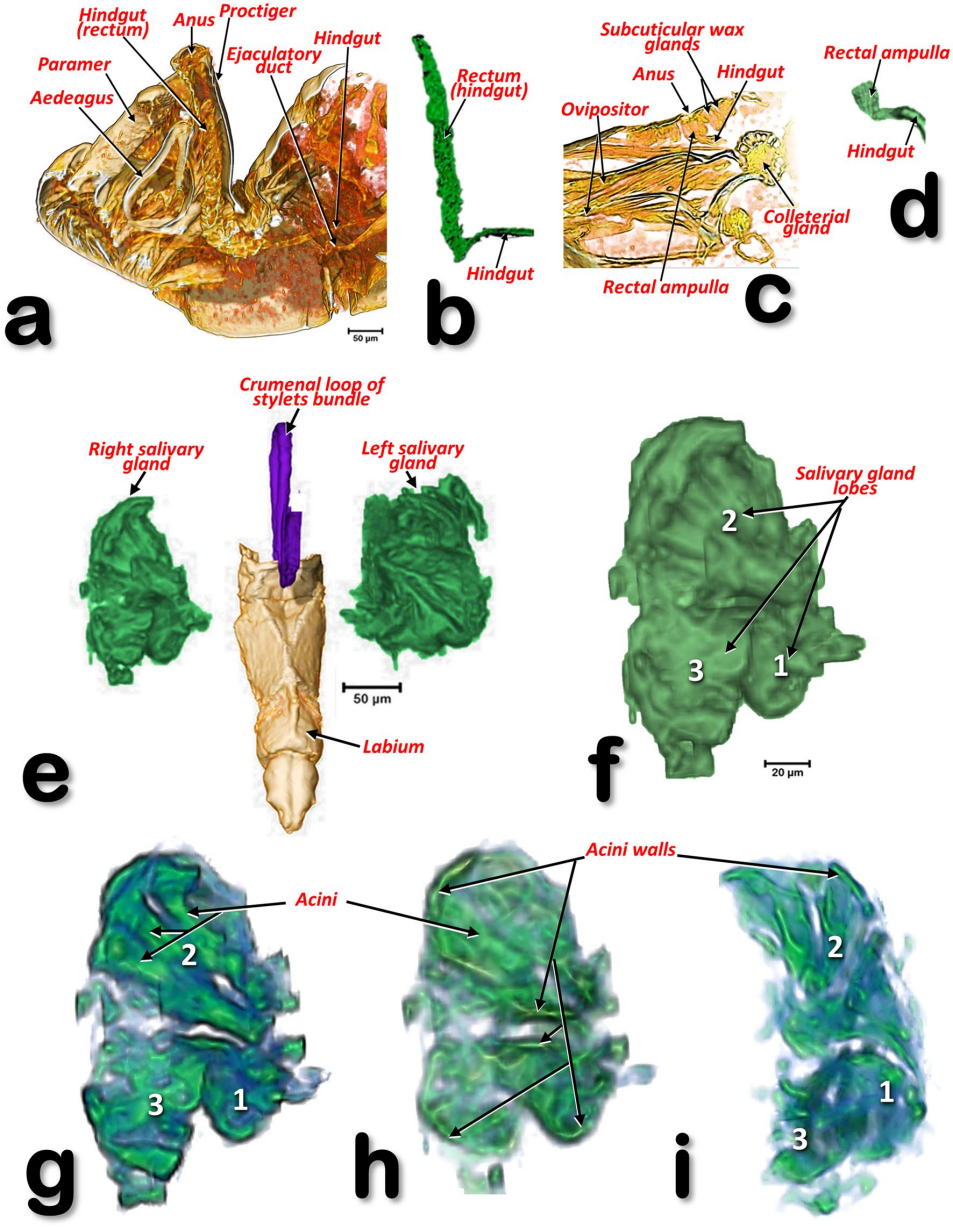
Volume-rendered images of the abdominal tips of a male *Diaphorina citri* (**a**, **b**) and a female *D. citri* (**c**, **d**), showing the rectal differences (**b**, **d**). Volume-rendered images of the salivary glands of a male *D. citri* in a frontal (**e**–**h**), and in an inner right-lateral views (**i**). Un-cleared (**e**, **f**), and cleared volume-rendered images (**g**–**i**). Slice ca. 7 µm thick (**h**).

**Figure 17:**
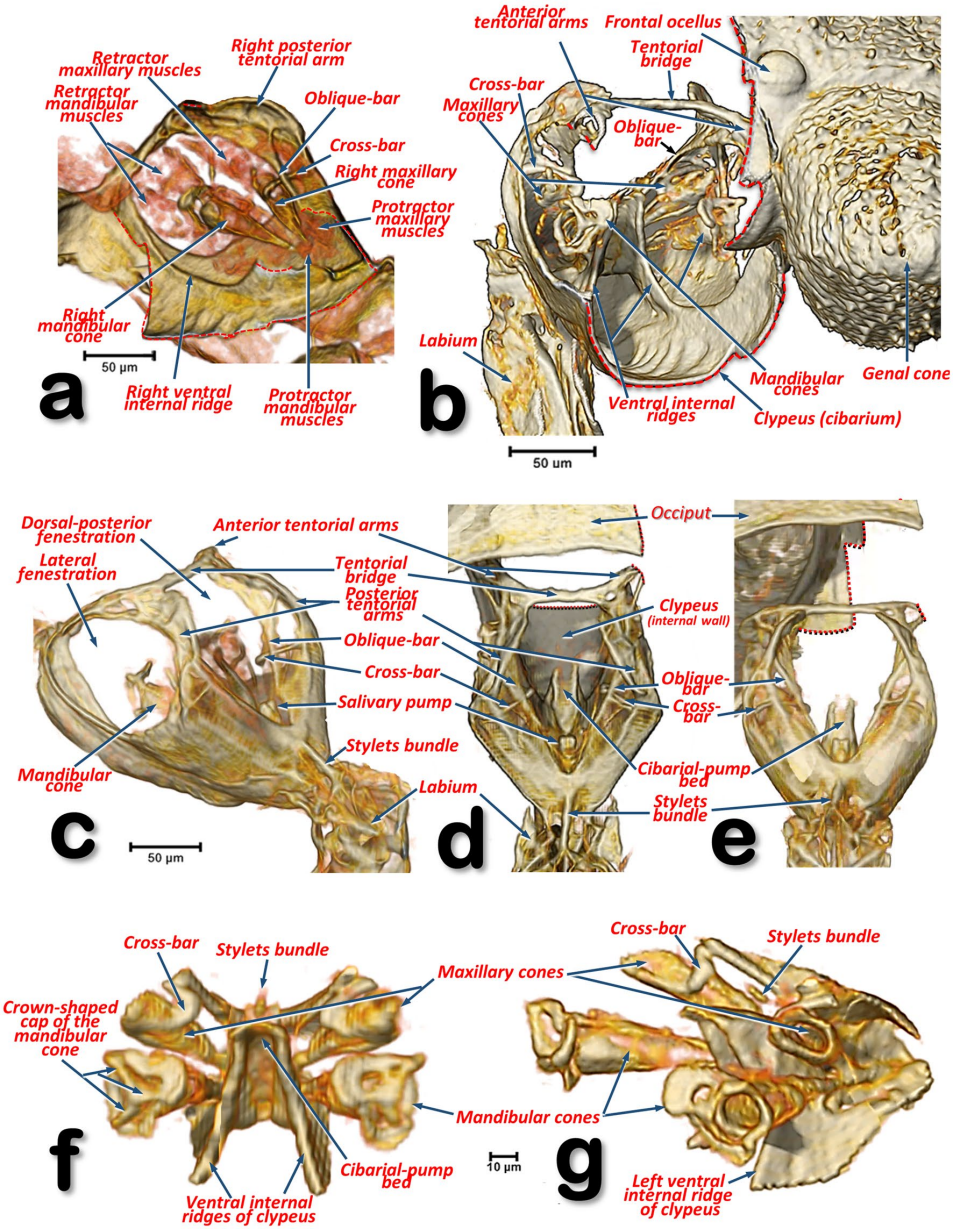
Volume-rendered detailed images of the feeding apparatus and tentorium of a male *Diaphorina citri* in different perspective views. Left-lateral (**a**, **g**), right-frontal (**b**), left-posterior (**c**), dorsal (**d**), posterior (**e**), frontal (**f**). Volume close-up images of the cibarium: internal right-side view, with the maxillary and mandibular cones rendered (**a**), and only the ‘harder’ structures (**b**–**g**). Details of the mandibular and maxillary cones (**f**, **g**). To be able to see inside the cibarium, different virtual cuts were made using software (cuts are indicated with dotted red lines).

**Figure 18:**
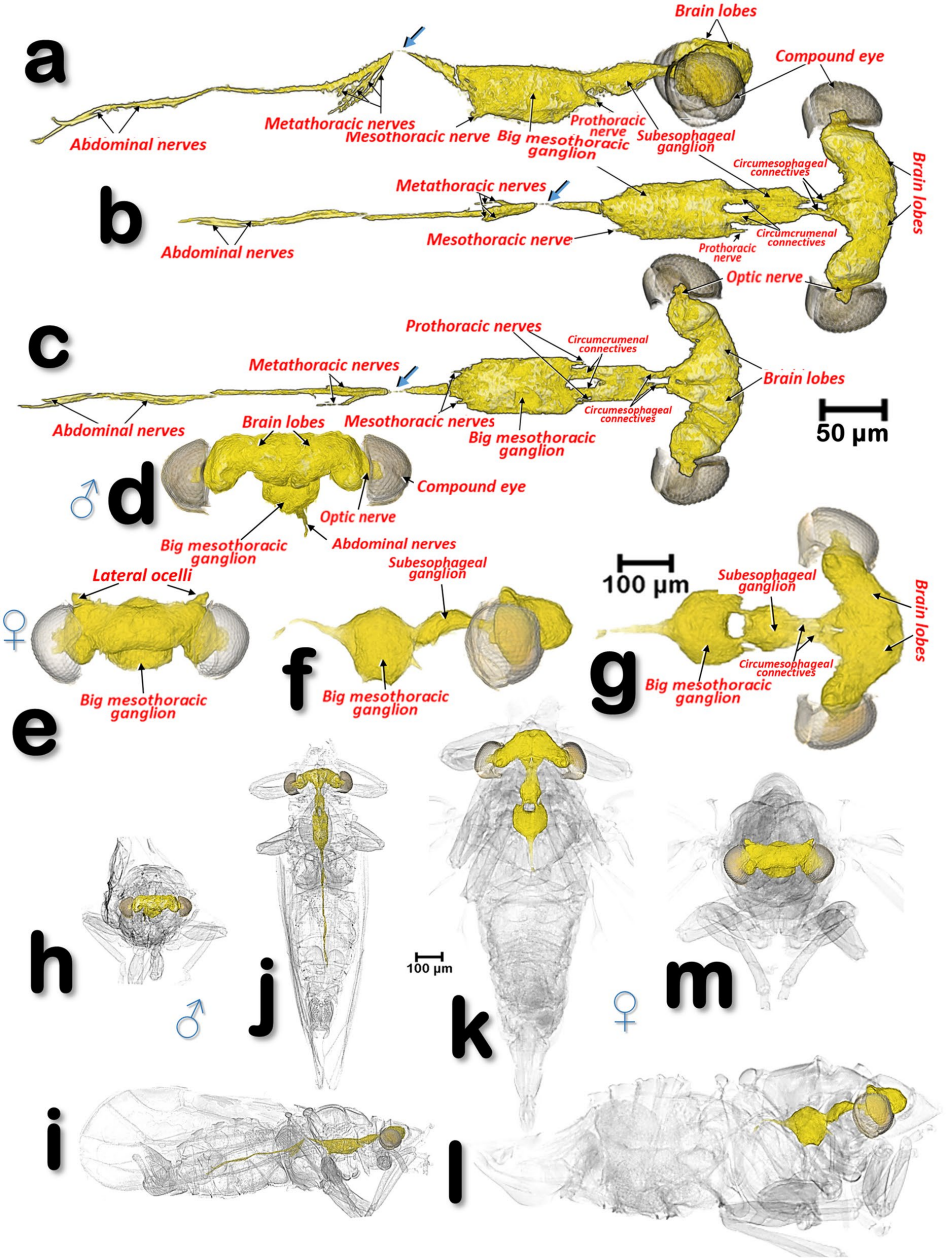
Volume-rendered images of the nervous system of a male *Diaphorina citri* (**a**–**d**, **h**–**i**), and a female *D. citri* (**e**–**g**, **k**–**m**). Views are: right-lateral (**a**, **f**, **i**, **l**), dorsal (**b**, **g**, **j**, **k**), ventral (**c**) and frontal (**d**, **e**, **h**, **m**). Blue arrows indicate a narrowing of the ventral cord just as it passes through the wall between the meso- and metathorax (**a**-**c**). Note that in the upper images (**a**-**g**) the scale for the male and female are not the same, and that in the female only the beginning of the abdominal nerves are rendered.

**Figure 19:**
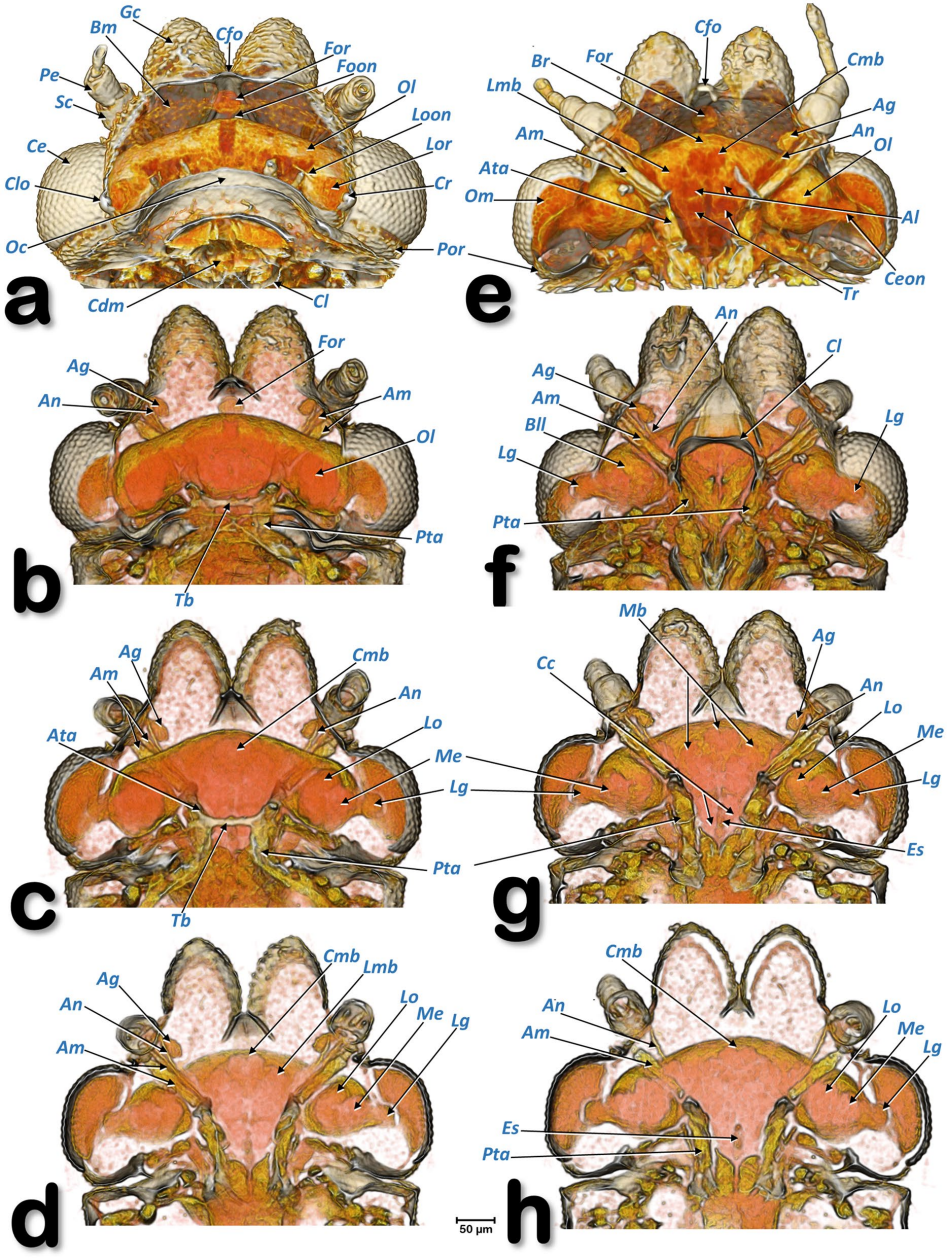
Volume-rendered images of the head of a female *Diaphorina citri* showing the brain and other internal structures. Dorsal views in the left-hand column (**a**–**d**) and ventral views in the right-hand column (**e**–**h**). From the top to the bottom successive dorsal/ventral cuts were made to obtain virtual slices. Abbreviations: *Ag* = antennal gland; *Al* = antennal lobe (deutocerebrum); *Am* = antennal muscles; *An* = antennal nerve; *Ata* = anterior tentorial arms; *Bll* = lateral lobe of brain; *Bm* = basal membrane; *Br* = brain (protocerebrum); *Cc* = circumesophageal connectives; *Ce* = compound eye; *Cdm* = cibarial dilator muscles; *Ceon* = compound eye optic nerve; *Cl* = clypeus; *Cfo* = cornea of the frontal ocellus; *Clo* = cornea of the lateral ocellus; *Cmb* = central mushroom body; *Cr* = crystalline lens; *Es* = esophagus; *Foon* = frontal ocellar optic nerve; *For* = frontal ocellar retina; *Lg* = lamina ganglionaris; *Lmb* = lateral mushroom body; *Lo* = lobulla; *Loon* =lateral ocellar optic nerve; *Lor* = lateral ocellar retina; *Mb* = mushroom bodies; *Me* = medulla; *Oc* = occiput; *Ol* = optic lobe; *Om* = ommatidia; *Pe* = pedicell; *Pta* = posterior tentorial arm; *Sc* = scapus; *Tb* = tentorial bridge; *Tr* = tritocerebrum.

### The respiratory system

This opens externally via three pairs of thoracic spiracles and seven pairs of abdominal spiracles (situated on the pleural region of the thoracic segments and the 1st-7th abdominal segments; the 1st and 2nd are very close to each other; Figs. 3a, 6a,d, 9a and Supplementary video S11). Using software to achieve transparency it was possible to highlight the abdominal tracheal tubular system of a female (Fig. 9b,c and Supplementary Video S1). This is organized as: a pair of dorsal longitudinal tracheal trunks, situated in parallel and following the dorsal vessel (Fig. 10b,c); a pair of lateral longitudinal tracheal trunks (located in the dorsal abdominal third), each with a double tube branching anteriorly and entering into the thorax; and ventrally a pair of longitudinal tracheal trunks. The dorsal longitudinal tracheal trunks and the lateral longitudinal tracheal trunks are interconnected by dorso-lateral segmental tracheae. The lateral longitudinal tracheal trunks and the ventral longitudinal tracheal trunks are interconnected by latero-ventral segmental and spiracular tracheae.

### The dorsal vessel (heart)

This is located in a dorsal-medial abdominal position, and situated immediately below the 4th-7th abdominal tergites (Fig. 10a). It has four dilated heart chambers each opened by a pair of lateral ostia (Figs. 9a, 10c and Supplementary Video S2). The dorsal vessel is extended forwards into an aorta located in an anterior position, starting at the third abdominal segment (Fig. 10b,c). Lateral to each heart chamber are fascicles of alary muscles, (delta-wing-shaped, Fig. 10b).

### The digestive system and associated structures

The ACP has a rather complex piercing and pumping feeding apparatus (labium, mandibular/maxillary stylets and a cibarial pump). The digestive system sensu stricto (Figs. 11, 12, 13, 14, 15 and Supplementary Videos S5–S9) opens to a pharynx running anteriorly inside the cibarium (clypeus) (Fig. 15f–h); the pharynx crosses the ventral nerve cord between the brain and the subesophageal ganglion and it has a precibarial-pump (the salivary pump) (Fig. 17c–d and Supplementary Video S4) and also a cibarial pump (Fig. 15c,h) with an internal flapping diaphragmatic cibarial valve (Fig. 15d). The cibarial pump is connected to four pairs of cibarial dilator muscles. Dorsally, each pair of cibarial dilator muscles join medially and appear v-shaped; the first pair of muscles are clearly the largest, decreasing in size from the 1st to the 4th (Fig. 15g and Supplementary Video S7).

#### The esophagus

The pharynx continues into the esophagus which, after a large loop, turn backwards, passing on and close to the ventral nerve cord (Figs. 11a,c, 12b and Supplementary Video S5), and then dilates slightly before connecting with the filter chamber (Fig. 15d,e and Supplementary Videos S5, S10).

#### The midgut

The esophagus connects with a dilated afferent midgut (crop), runs backwards to the right and immediately down to form the medial descending midgut arm. After a ventral midgut loop the ascending midgut arm goes left and dorsally, and, after a fore dorsal midgut loop, a right fore midgut dorsal arm runs backwards and down to a right descending dorsal middle arm; from here and after a right midgut middle loop, is the start of the right ascending midgut dorsal middle arm in which the 1st midgut appendage is inserted (located in a right-fore position); after this, the right hind midgut dorsal arm continues and joins the 2nd midgut appendage (located in a right-hind position); after this, the left hind midgut dorsal arm continues forwards (the 3rd midgut appendage connects to it and is located in a left-hind position immediately after the connection of the 2nd midgut appendage), and immediately turns down, forming a short left descending midgut dorsal arm which, after a left midgut middle loop, turns upwards forming a short left ascending midgut dorsal middle arm, and then the afferent midgut; the midgut connects to the filter chamber in a posterior dorsal position. The walls of the midgut have very conspicuous rhomboid cells (Fig. 13a and Supplementary Video S8).

#### The appendages of the midgut

The four appendages show a parallel symmetrical arrangement in pairs, with the 1st and 4th directed forward and the 2nd and 3rd directed backwards (Figs. 11, 12, 13a–c and Supplementary videos S5, S6, S8). They show a tubular structure; the walls are formed from a single layer of cells delimiting an internal lumen (Figs. 11d,e, 13, and Supplementary Videos S5, S6, S8).

#### The inner hindgut

After the midgut, a narrower inner hindgut continues inside the filter chamber; it turns one way and forms a U-turn on the right side, makes two complete turns and exits the filter chamber on the front left side, just behind the esophagus insertion. The inner hindgut progressively increases in diameter; by the last turn it has doubled in diameter, but then thins out as it leaves the filter chamber to form the outer hindgut (Fig. 14 and Supplementary Video S9).

#### The filter chamber

This is located in the anterior third of the abdomen, in a dorsal-central position and partially extending into the 3rd abdominal segment (Fig. 12 and Supplementary Video S6). It is inclined at an angle of circa 45º with the anterior part (where the esophagus joins) at the same level as where the thorax separates from the abdomen, and lower than the posterior part (Figs. 11 and 12, Supplementary Videos S5 and S6). Within the chamber there are three separate regions, or spaces, enclosed in a sheath and surrounded by the inner hindgut. The three regions can be distinguished from each other: the anterior region is spongy, pyriform (1, bulb) and more voluminous than the others; then there is an intermediate region (2) and a posterior region (3) that are of progressively smaller volumes (Fig. 14c,d and Supplementary Video S9).

#### The outer hindgut

The outer hindgut begins at the upper left side of the filter chamber very close behind the point where the esophagus joins (Figs. 13b,d,e, 14a,b,g,h and Supplementary Video S9). It runs above the nerve cord along the length of the abdomen ultimately connecting with the anus (Figs. 11a, 12a and Supplementary Videos S4, S5). The hindgut is posteriorly differentiated into a rectum that, in males, is configured as a long, flared tube; in females it forms a small rectal ampulla, just below the anus (Figs. 11a–c, 12, 16a–d and Supplementary Videos S5, S6).

#### Salivary glands

The salivary glands are located anteriorly and on both sides of the thorax (Figs. 11a–c, 16a and Supplementary Videos S5–S6). They are vertically arranged in a ‘C’-shaped configuration (concave anteriorly) each with three main lobes; each lobe has an acinar configuration with converging bunches of acini (Fig. 16f and Supplementary Video S10).

#### Cibarial structures

The internal anatomical details of the cibarial structures are shown in Fig. 17 and Supplementary Videos S3, S4. The cibarial space has three large openings, or fenestrations (one dorsal and two laterals), and the walls are reinforced dorsally by the tentorial bridge bar and posteriorly by prolongations of the tentorial arms, forming an oblique bar and a cross-bar. Moreover, there are two ventral ridges giving support posteriorly to the cibarial-pump bed.

#### The maxillary and mandibular structures

The maxillary and mandibular cones are inside the cibarium (Fig. 17a–c,f,g and Supplementary Video S3). The maxillary cones are dorsal to the mandibular ones, and connected to the cibarial cross-bar. The maxillary/mandibular retractor/protractor muscles are shown in Fig. 17a. The mandibular cones have a crown-shaped cap (Fig. 17f,g and Supplementary Video S3). Stylets emerge from the basal apex of the cones, meeting together in a stylet bundle. Close to the base of the cones, where the stylets emerge, is a small precibarial salivary pump (Fig. 17c–e). The stylet bundle can be retracted back and stored in the crumenal pouch; when stored, this forms a characteristic crumenal loop (Figs. 11a, 12b, 15 and Supplementary Videos S5, S7). This loop passes through the nerve cord behind the subesophageal ganglion (Figs. 11a, 12b and Supplementary Videos S5–S7).

### The nervous system

The nervous system and its spatial relationship with the digestive system is shown in Figs. 11, 12, 15a–c and Supplementary Videos S5, S6. Detailed views of the nervous system in both sexes are shown in Fig. 18, and detailed views of the brain within the head are shown in Fig. 19. The brain has two lateral lobes (each consists of three successive neuropiles: the lamina, the medulla and the lobula complex) forming the optic nerves that connect with the compound eyes (Figs. 18b–d,g, 19 and Supplementary Video S11). Anteriorly within the brain there is one central and two lateral mushroom bodies with poorly marked calyces (Fig. 19c,d,g,h). Two circum-pharyngeal connectives surround the pharynx and connect with a subesophageal ganglion (Figs. 11a, 12a,b, 18b,c,g). The subesophageal ganglion is connected by circumcrumenal connectives to a large, mesothoracic ganglion. Exiting at each side of the mesothoracic ganglion are a pair of prothoracic nerves (anteriorly), and a pair of mesothoracic nerves (posteriorly) (Figs. 12b and 18a–c,f,g). The large mesothoracic ganglion is connected to a ventral cord that becomes narrower at the junction of the meso and metathoracic segments. In the metathorax, at the level of the meta-coxae, several ventral meta-thoracic nerves appear, and continue via two abdominal nerves along the length of the abdomen (Figs. 11a, 12b, 18a–c and Supplementary Videos S5, S6).

### Tentorium

The cephalic endoskeleton (tentorium) is shown in Figs. 17a–e, 19b–h, Supplementary Videos S3 and S4.

### Antennal glands

This is inside the cephalic capsule (head) and there is a sac-like antennal gland connected to each antennal scapus (Fig. 19b–g). Details of the interior of the cephalic capsule including the brain and eyes are shown in Fig. 19 and Supplementary Video S11.

### Feeding apparatus

A male feeding on a citrus (orange) leaf is shown in Figs. 6d, 7, 8a and Supplementary Video S3.

Penetration of the salivary flange is clearly visible (Figs. 7a,b, 8a,b), as well as the stylets and salivary sheaths that are inside a vascular bundle of the leaf and reaching the phloem (Figs. 7a,b, 8a and Supplementary Video S3). Three different shaped salivary sheaths were observed: branched (Figs. 6d, 7a,b, 8b and Supplementary Video S3), rosary-beads (Fig. 8c,d) and linear (Fig. 8c ). The presence of salivary sheaths resulting from failed piercing/sucking attempts are shown in Figs. 6d and 8c.

### Structure of the citrus leaf

Anatomical structural details of the citrus leaf can be observed in Figs. 6d, 7a,b, 8 and Supplementary Video S3.

## Discussion

The terminology we have used for external anatomy maintains the terms most widely used and follows classical entomological papers^33, 40, 45, 53, 61–64^ and specific psylloid publications^9, 17, 38, 39, 65^. The adult antennal olfatori sensillae of *Homotoma ficus* were recorded in the XIXth century as ‘odour pits’ (*Geruchsgruben*)^5^, and described as present in the flagellar articles, just as we, and other authors^41, 48^, have observed them in ACP. The olfatory function of these structures was assumed and, therefore, the term rhinaria frequently used to describe them (e.g.^37, 38, 48, 65, 66^). Using SEM it has been possible to confirm that these rhinaria correspond to campaniform sensillae^41, 48^ and by investigating odorant-binding proteins, odor receptors and odour-degrading enzymes, their function as odour receptors has been demonstrated^41^.

Within the feeding apparatus, the labium of *Psyllopsis fraxinicola* has been described and visualized previously by Witlaczil^5^ as a three segmented structure. This author also drew schematics of the maxillary/mandibular cones inside the cibarium and the associated protractor/retractor muscles; his findings fit with our results for ACP and also with a detailed SEM study^18^ describing the existence of a posterior slit on the 3rd labial segment. In another study an association between this posterior slit and a dorsal groove was described^9^. However, we observed a long anterior (dorsal) dividing slit, rather than a groove^9^. The 3rd segment of the ACP’s labium has a configuration very similar to that seen by SEM in *Cacopsylla chinensis*^27^ and the melaleuca psyllid, *Boreioglycaspis melaleucae*^67^.

In Hemiptera the labium is also known as the rostrum^64^. As a simplification, the term rostrum has also been used by Cicero and colleagues to describe the external, ventral aspect of the ventralized portion of the head, i.e. a consolidation of the clypeus, postclypeus, anteclypeus and labrum described by classical authors^9, 13^. However, to avoid uncertainty inherent in attempts to homologize structures^68^ and avoid confusion, we have used the term clypeus, rather than rostrum, to refer to the ventral bulging structure of the head which encloses the cibarium. We have chosen this because this is the term most commonly used for that structure in homopteran insects^40, 45^ and specifically in psyllids^23, 27, 43, 46, 55, 65, 67^.

For *Psylla mali*, a number of studies have included realistic drawings of the feeding apparatus and the maxillary/mandibular cones inside the cibarium^23, 43, 46, 55^; these have a very similar configuration to that which we describe here for ACP. More recently, there have been several studies on the structures inside the cibarium of several species^10, 11, 13, 27, 56^, including ACP^9, 14, 18^. Although all these studies were impressive, they were limited because the structures described had to be dissected from the insect and subjected to preparation processes such as transparency digestion with KOH; this caused distortion and influenced the results obtained. For instance, the typical crown-shaped cap of the mandibular cones (observed also in other species e.g. *P. mali*^46^) were missing in SEM images, appearing only as a medial expansion of the cap that the authors termed the auricle^10, 13, 14, 56^.

Detailed studies of the thorax of various psylloid species have been published^17, 39, 43, 46, 55^ but, to our knowledge, there have been no detailed studies of the thorax of ACP .

Leg glands, and particularly coxal glands, have been described in other insect species, e.g. hymenopterans^69–73^ but, to our knowledge, not for any psylloid species. However, in published SEM images of species in the genus *Cacopsylla*^17^ coxal orifices (ostioles) are clearly visible, just as we observed them in ACP.

Antennal glands have been reported in other insect species including ants^74–76^; Dams observed antennal glands in the scapus of males from the hymenopteran species *Melittobia australica* and concluded: “Presumably males rely on chemical and tactile stimuli for locating females and for precopulatory behaviour. Chemical information appears to play an important role in the behaviour of both sexes. The size of the complex gland in the male scape and the role of this segment during precopulatory behaviour suggests that the female receives a considerable chemical input during antennation”^77^. Moreover, males of ACP are known to be attracted to a volatile pheromone produced by females, and to colonize citrus plants that were colonized, or had been previously colonized, by females^78^. However, the location for pheromone production has not been identified. Considering the basal location of the coxal glands, it is quite plausible that, if they produced pheromones, they would be readily transferred to plant surfaces during colonization. It is also possible that the antennal glands could be involved in the production of chemicals that aid recognition during close-range courtship. In fact, in the recent revision of ACP mating behaviour by Mankin and Rhode^79^, short-range semiochemicals were shown to play a role in mate-finding in several, but not all, psylloid species. Future research should investigate the secretory role of these structures and, where appropriate, the chemical composition, function and use of the compounds produced as attractants.

There are some discrepancies in the literature concerning the interpretation of wing venation (e.g. between the general publication on psyllids by Hodkinson and White^65^ with the publication of Ossianilson^38^). We followed Yen and Burckhardt^47^ for assigning names to the veins of the fore wings and Ossianilson^38^ for the hind wings. Venations of the wings are characteristically tubular structures, clearly visible in cross-section. The structure generally known as the Cu_2_^38, 65, 80^ is not tubular and cannot be considered as a vein; it is in fact a fold on the cubital area. In fact, at the end of the XIXth century Mally^29^ considered it to be a suture, the ‘claval suture’. We name it the cubital fold but, to aid recognition in the figures we have retained the abbreviation Cu_2_ in parenthesis.

In psyllids the first abdominal segments are reduced, and are joined to the segments of the thorax or abdomen. This makes it difficult to determine how many of them there are. Thus, to date there has been no consensus concerning the visible sclerites of the abdominal segments^32, 36, 40, 49, 53^. In fact, many publications on psyllids avoid the controversy and do not number these segments e.g.^65, 80^.

There are issues with interpretation of the abdominal segment sclerites but, as this is not the focus of this paper, we have adopted the numbering system for tergites and sternites that fits a (difficult) general consensus, but particularly Matsuda’s interpretation^32^. The results we present here complement those we have already published of the abdominal anatomy and genitalia (terminalia) of male^8^ and female ACP^7^.

In psylloids, the small size, and sometimes cryptic position, of some respiratory spiracles has resulted in controversy about their number and location^25, 36^ but, after an extensive revision, the conclusion was that all psyllids have three pairs of thoracic spiracles^25^ and specifically: “so far as can now be ascertained, no psyllid possesses more than seven functional pairs of abdominal spiracles, although the possibility of an eighth pair, particularly in the male, is not overlooked”. This agrees with our observation of seven pairs of abdominal spiracles in ACP; we did not find an eighth pair. Moreover the 1st and 2nd abdominal pairs of spiracles are very close to each, as previously observed for *P. mali*^36^.

The tubular tracheal system of psylloid species has not previously been studied and described in detail, particularly for adults. Witlaczil^5^ described and presented images of this in a nymph of *Trioza rhamni*, but recorded that it was difficult to observe in adults; he also included a schematic diagram of the dorsal view of the thoracic tracheal tubes of *P. fraxinicola*, and a lateral view of the thoracic tracheal trunks of *Rhinocola speciosa*. Similarly, a recent paper using the lactic acid clearing technique provided visual evidence, but not details, of tracheal tubes in a nymph of *Cyamophila willieti*^81^. To our knowledge, our results on the abdominal tracheal tubular system of ACP represent the most detailed study to date. In the future the micro-CT technique described for coffee berry borer beetle^82^ could be used to study the ACP respiratory system in even greater detail. However, the micro-CT technique scans specimens that are either anesthetised or have just been killed, i.e. requiring work on living insects; as ACP is an invasive pest and there is a potential risk of escape, this would represent a difficulty for us in Spain.

The circulatory system of insects is responsible for movement of the haemolymph into the haemocoel spaces where organs are immersed. The haemolymph enters various contractile chambers of a dorsal vessel (heart) and is pumped out through an anterior aorta. This is considered as an open circulatory system; it does not play an important role in the transport of gases which is achieved by an aero-vascular system formed from a complex of tubes (the tracheal system)i.e.:^45, 63, 64, 83^. The heart and anterior aorta was first studied and images presented by Witlaczil^5^ in two psyllid species; even though his drawings were largely schematic, descriptions of the abdominal position and extension of the heart in *R. speciose*, and the extension and lateral ostioles of *Psylla buxi*, are in full agreement with our results for ACP. Thus, to date, our results represent the most detailed study of the tracheal system of psylloids, and the only one for ACP.

The alimentary canal (digestive canal sensu stricto) of psylloids including ACP, is, with the external anatomy, the most studied and visualised organ^5, 9, 11, 50, 51, 55, 56, 84–87^. Until the present study, the most detailed studies on adult ACP were those of Cicero et al.^9, 11, 56^, based on dissections and images taken using optical and electronic microscopy, which allowed the production of schematic drawings that summarized the general organisation of the alimentary canal.

Snodgrass^45^ described the typical homopteran alimentary canal as having a ventriculus consisting of three parts: 1st ventriculus (an anterior enlargement lying immediately posterior to the stomodaeal valve and enclosed in the filter chamber); 2nd ventriculus (a croplike sac serving as a storage reservoir); and the 3rd ventriculus (a long, tubular section, the functional stomach of the insect, which turns anteriorly to re-enter the filter chamber). Psylloids including ACP all show this organisation, specifically we found: the 1st ventriculus comprised of a bulb and two spongy spaces inside the filter chamber; the 2nd ventriculus comprised of the dilated efferent midgut (‘crop’), with a transitional descending midgut ventral arm (in which the rhomboid cells are very conspicuous as they are in the efferent dilated midgut); and the 3rd ventriculus after the ventral midgut loop and continuing on to the inner hindgut inside the filter chamber. This was similar to previous descriptions and figures^9, 11, 56^. However, as a result of dissection and associated manipulation, some the anatomical positions of structures in these earlier studies do not correspond with the actual arrangement inside the insect as we observed using micro-CT. For instance, it was stated that “the esophagus enters the filter chamber vertically”, but with micro-CT we see that the esophagus descends and turns almost horizontally at the dilated end before entering the filter chamber. Similarly, earlier studies found that the 1st ventriculus (inside the filter chamber) was formed from an ‘upper’ (the bulb) and a ‘lower’ ventriculus, but micro-CT shows that the bulb is anterior and situated in a lower position compared with the posterior part of the 1st ventriculus.

In general, the alimentary canal of insects starts with the mouth, ends with the anus and consists of three main parts: foregut (stomodaeum), midgut (mesenteron) and hindgut (proctodaeum). In contrast with the midgut, the foregut and hindgut are ectodermic derivates and are chitinized. The foregut consists of the preoral cavity, the mouthparts, the pharynx and the esophagus. From here, food enters the midgut and eventually the hindgut. Waste is excreted through the posterior opening (anus) of the alimentary canal^45, 63, 64^. If we follow this general scheme with the corresponding terms for ACP, the mouthparts correspond to the mandibular and maxillary stylets that are protected inside the labium and together form an alimentary canal^9, 18^. Adaptation to a piercing/sucking feeding strategy means that what should strictly be considered as the preoral cavity and mouth is controversial in homopterans^9^. The pharynx corresponds to the anterior part of the alimentary canal, it has a small enlargement, containing the precibarial salivary pump (our observation of this in ACP had a similar configuration to that described and visualized for *P. mali* by Grove^23^ and Weber^46^, and for *Bactericera cockerelli* by Cicero et al*.,*^13^), and a further bigger enlargement which, together with the dilator muscles, formed the cibarial pump. The midgut extends from the esophagus and dilates just before connecting with the filter chamber; after the filter chamber the midgut continues ultimately diminishing in diameter and re-entering the filter chamber as the hindgut (inner hindgut). The hindgut then exits the filter chamber as the outer hindgut, which then dilates into a rectum just before the anus.

The structure of the rectum varies between the sexes. In males the rectum is a long uniformly dilated tube while in females it ends in a rectal ampulla. This could be explained by variation in defecation behavior between the sexes, as has been described previously for the melaleuca psyllid, *Boreioglycaspis melaleucae*. In *B. melaleucae*, adult females produce whitish honeydew balls that are powerfully propelled away from the body, probably to ensure these sticky excretions do not adhere to nearby eggs and newly-hatched nymphs. In contrast, adult males produce clear droplets of honeydew that are not propelled away from them^67^. It is likely that ACP females also propel their droppings away from themselves to avoid contaminating progeny; the rectal ampulla observed in females would facilitate it much better than the more voluminous long rectum seen in males.

Ammar et al*.,*^19^ distinguished three sections in the midgut of ACP and we observed the same: an anterior-midgut extending from the point of contact with the filter chamber until the position where the 1st abdominal appendage connects (we have termed this the dilated efferent midgut); a middle-midgut that extends from where the 1st abdominal appendage connects until the point at which the 4th abdominal appendage connects; and a posterior-midgut that extends from the insertion of the 4th abdominal appendage until connection with the filter chamber (we have termed this the afferent midgut).

The filter chamber occurs in most homopteran insects and is a modification of the alimentary canal in which two ordinarily distant parts of the digestive tube (the stomodeum/mesenteron and the proctodaeum) are bound adjacent to each other by a connective tissue sheath. The constituent parts of the filter chamber are usually the two extremities of the mesenteron and the anterior end of the proctodaeum. Thus, the organ formed is considered to be a device for allowing some of the excess water and soluble carbohydrates from food to be eliminated by diffusion directly from the anterior part of the digestive canal into the posterior; the protein and fatty materials are retained to be digested and absorbed in the stomach^45^. The alimentary tract within the filter chamber consists of two connected sections with luminal flows moving in an antiparallel direction that, together, represent a counter-current filter^88^. To function this filter requires the liquid within to be under high pressure, which explains why the esophagus is dilated just before it enters the filter chamber, as we observed, and has been described previously^9, 11, 86^; according to the Bernoulli principle an increased pipe diameter means a decrease in velocity and kinetic energy and an increase in pressure^89^. Thereafter, the liquid food is forced under pressure into the filter chamber where the speed slows to facilitate transfer of excess water into the inner hindgut that surrounds the filter chamber. It seems that dilation of the esophagus before entry into the filter chamber occurs in many sap-sucking homopterans, thus this enlargement is clearly visible in the figures in numerous publications e.g.^40, 45, 90^.

The filter chamber has been studied in detail in a range of homopteran species, and more than 50 papers on its anatomy and physiology have been published e.g.^86, 91–95^. In psylloids the hind-gut and oesophagus are spirally wound round one another^40, 90^, and have been described previously for *P. mali*^43, 55^ and *P. fraxinicola*^5^. With respect to ACP light microscopic images of the digestive tract, including the filter chamber, have been published and there are general schematic anatomical interpretative drawings with a SEM image^11^, and a micrograph of the filter chamber of one last instar nymph^56^. Moreover, the anatomy of the alimentary canal of ACP, including the filter chamber, has been recently summarised as animated schematic drawings^86^.

The four abdominal appendages that end at different points along the midgut are widely considered as Malpighian tubules with an excretory function^5, 19, 40, 43, 50, 85, 88, 96^. From an anatomical point of view, the insertion of Malpighian tubules should be coincident with the end of the mesodeum (midgut) and beginning of the proctodaeum (hindgut)^45, 63, 64^. However, the appendages of the midgut flow into the midgut consecutively and are widely separated from each other. This is why we decided to follow Cicero’s terminology and use the term abdominal appendages rather than Malpighian tubules, until any potential excretory function is elucidated^9, 11, 56^. Micrograph figures of the Malpighian tubules of the bee *Melipona scutellaris*^97^ are fully comparable with our micro-CT volume-rendered images of the midgut appendages of ACP, and also similar to structures described for the psyllids *Psylla alni* and *T. rhamni*^5^ and termed Malpighian tubules.

There are several papers that describe and/or provide images of the salivary glands of potato psyllid *B. cockerelli*^13^ and ACP^9, 11, 19, 85, 98^. The salivary glands of adult ACP are described as each being comprised of two principal salivary glands and a smaller accessory salivary gland. Comparing these descriptions and micrographs with our results we find that what were described as principal salivary glands are equivalent to the salivary lobes we observed with micro-CT and numbered lobe 1 and lobe 2; what we describe as lobe 3 corresponds with what was previously described as a separate accessory salivary gland. Independently of any observed structural/content differences (see^11, 19^), all three lobes together form a lateral salivary gland, and we see no reason to separate the 3rd lobe as a different separate accessory salivary gland. Using micro-CT we observed bunches of acini in each lobe and this is in full agreement with previously published images taken from a semithin section (stained with toluidine blue) of the principal salivary gland which showed at least three differentially stained acini/groups of secretory cells^19^.

The anatomy of the nervous system in insects has been widely studied, including diagrams made more than 400 years ago by Aldrovandi^99^ and Malpighi^100^. Since then, many general descriptions of insect nervous systems have been published e.g.^45, 63, 64, 90, 101^, including for homopterans^40^. Recently micro-CT has been used to study the anatomy of the nervous system e.g.^58–60, 102–109^. Anatomical studies with images of the nervous system have been made for a range of psylloid species^5, 29, 43, 46, 49, 55, 110^, including ACP^19, 60, 85^. The results we describe here are the first complete study of the nervous system of ACP, and the general organisation conforms with existing descriptions for psylloids.

The ACP brain has the same general organisation as other insects where the brain represents the main association centre. It includes the protocerebrum, deutocerebrum and tritocerebrum. The protocerebrum, the l contains both neural and neurosecretory elements. With anterior ocellar nerves, and lateral optic lobes. A pair of association centres, the corpora pendunculata, also known as the mushroom bodies, are important association centres, by especially receiving both sensory olfactory and visual inputs. They are considered to play an important role in learning and memory, and in social Hymenoptera their size has been correlated with the extremely complex behaviour patterns of these insects. There are three neuropilar masses (lobulla, medulla and lamina ganglionaris) in each optic. The deutocerebrum contains the paired antennal lobes, that also play an important role in learned and olfactory behaviour. Beneath the deutocerebrum is located a small structure, the tritocerebrum, with sensory and motor functions^45, 63, 64^.

Previously, the large mesothoracic ganglion has been called the thoracic ganglion^43, 55^. We have named this the big mesothoracic ganglion to distinguish it from the subesophageal ganglion, and we agree that this ganglion is likely to be an amalgamation of thoracic and abdominal ganglia, as has been reported for other hemipterans^19^. In fact, in other insects the abdominal ganglion is fused with ganglia of the thorax, to form a single ganglion; this has been named the thoraco-abdominal ganglion^64^.

Sexual differences in the nervous system of insects have been reported, both in morphology and size, and in structural neuron connections (e.g. in social insects^111^, flies^112^ and beetles^107^). Here we report on sexual differences in the nervous system of ACP for the first time. Males have a slender configuration of the brain and almost parallel margins of the subesophageal and large mesothoracic ganglion, while in females both brain and ganglia are more voluminous, and the large mesothoracic ganglion is almost spherical in shape.

The ACP is a phloem-feeding, sap-sucking hemipteran species. Its feeding behaviour has been investigated using the electrical penetration graph (EPG) technique^113, 114^ and light microscopy has been used to confirm that the stylets reach the phloem (this also included observations of the salivary sheath tracks)^114^. Different methodologies have been used to study homopteran salivary sheaths inside the plant tissues (e.g.^35, 96, 115, 116^). The micro-CT technique has been used to visualise stylet penetration and the salivary sheaths of the meadow spittlebug *Philaenus spumarius*^117^ and the aphid species *Myzus persicae*^118^. The present study on ACP obtained rendered images that were fully comparable to previous images obtained using a variety of different techniques; our images include observations of the different configuration and typologies described previously and benefited from being high quality 3D images and videos.

All stain/contrast methodologies we used in the present study enhanced our views of structures. However, the best results were obtained by first feeding animals with orange plant material that had been submerged in BAPC (***Branched Amphiphilic Peptide Capsules***) linked to Hg as a contrast agent ^119^; using iodine enabled us to achieve the highest contrasted samples.

## Conclusions

The use of micro-CT techniques for elucidation of the anatomical structures and organs of ACP has enabled us to make a complete reconstruction of the anatomy of this insect, indicating the actual position of internal structures and organs without distortion resulting from dissection. We also include detailed rendered images and videos. This work represents the first complete micro-CT reconstruction of the anatomy of *D. citri*, which, together with our previous study on the male^8^ and female^7^ reproductive systems and bacteriome, is the first complete, detailed anatomical study of a psylloid. In general, it is the first micro-CT anatomical study of a hemipteran studied as a whole. Together with our previous papers on the coffee berry borer beetle^82,107^ these studies represent anatomical reconstructions of the smallest insects carried out to date using micro-CT. Moreover, this is the first report of coxal and antennal glands with a plausible pheromone secretion role, and the first report of differences between the sexes in internal anatomy (i.e. the hindgut, rectum, large mesothoracic ganglion and brain). Additionally, this is the first time a male feeding within an orange leaf has been reconstructed which enabled us to see the arrangement of the feeding apparatus (labium, stylets bundle), salivary flange, salivary sheaths; we were also able to see how the stylet bundle appears inside the cells of the citrus leaf, the penetration point of the salivary flange at the leaf surface, intact salivary sheaths and abandoned salivary sheaths that resulted from failed piercing attempts.

With the supplementary videos and 3D model of a male feeding on a citrus leaf (suitable for use on mobile devices), this package is useful for future research and/or for teaching insect anatomy to students and the general public. Together this constitutes a unique atlas of the anatomy of the ACP.

## Methods

Six adult female and five adult male ACP specimens were scanned for evaluation in this study. They were from the rearing facilities at the United States Department of Agriculture, Agriculture Research Service, Fort Pierce, Florida (USA). Four different staining/contrast methods were used:

1. Live psyllids were prepared by overnight fixation in 4% glutaraldehyde with 2.5% paraformaldehyde in sodium cacodylate buffer pH 6.5; the fixed samples were rinsed three times (10 min each) in 30% ethanol, then dehydrated in an ethanol series (30 min per step; 50%,70%, 80%, 90%, 95%) ending with three steps in 100% ethanol; finally, the samples were chemically dried by placing in 2 ml of 100% hexamethyldisilazane (HDMDS) for 2 h, followed by drying overnight at 35ºC. These specimens are visualized in Figs. 1, 2, 4e–g. .
2. Live psyllids were killed and preserved in 70% ethanol, then submerged in a 1% solution of iodine in 100% ethanol for 48 h; samples were transferred to HDMDS for 3 h and dried overnight at room temperature. These specimens are visualized in Figs. 3, 4a–d, 5, 9, 10, 15, 19 and Supplementary videos S1, S2, S11.
3. Psyllids were fed for 72 h on an orange sprig that had been submerged in Iomeron (iomeprol) contrast agent. This is a tri-iodinated non-ionic contrast media with a high concentration of iodine (400 mg/ml). We observed that the contrast medium was significantly absorbed into plant cuttings but that, even when diluted at 1%, it caused plant wilting, so it was necessary to dilute it to 0.1%. While in the act of feeding on citrus leaves the psyllids were flash frozen in liquid nitrogen, and then processed as described previously^120^. Finally, a small drop of fingernail polish placed on the posterior end of the psyllid and the leaf, was used to hold them in place after processing and drying. These specimens are visualized in Figs. 6, 7, 8, 17, Supplementary videos S3, S4, and Supplementary 3D model S12.
4. Adult psyllids were fed for three days on an orange tree sprig submerged in BAPC (***Branched Amphiphilic Peptide Capsules***) linked to Hg as a contrast agent ^119^*.* The insects were rinsed three times in 30% ethanol (10 min for each rinse), dehydrated in an ethanol series (30 min per step, 50%, 70%, 80%, 90%, 95%, and three times at 100%), chemically dried by submersion in 2 ml of 100% HMDS for 2 h, and dried overnight at 35ºC. These specimens were visualized in Figs. 11, 12, 13, 14, 17, 18 and Supplementary videos S5–S10.

Finally the specimens were prepared for scanning by gluing them to the tip of a nylon fishing line (200 µm in diameter) with cyanoacrylate, as previously described ^60, 121^. The prepared insects were scanned with a SkyScan 1172 desktop high-resolution micro-CT, with a Hamamatsu L702 source and a Ximea 11Mp camera. We used the following setting parameter ranges; isotropic voxel size = 0.52–0.54 µm per pixel; source voltage = 44-50KV, source current = 43-68µA, image rotation step = 0.2–0.5º, 360º rotation scan, binning 1 × 1, and no filter.

Recent versions of the Bruker micro-CT’s Skyscan software (NRecon v.1.7.4.6, DataViewer v.1.5.6.2, CTAnalyser v.1.18.8.0, https://www.bruker.com/products/microtomography.html) were used for primary reconstructions and the ‘cleaning’ process to obtain datasets on ‘slices’ through the insect as described previously^121^. The most recent version of the software CTVox v.3.3.1 (Bruker micro-CT’s Skyscan) was used to obtain the volume-rendered images seen in Figs. 1, 2, 6 and 7. Amira software, v. 2019.3 (Thermo Fisher Scientific, Waltham, MA)^122, 123^, with the built-in ‘volrenRed.col’ colour filter, was used to obtain the volume-rendered images seen in Figs. 3, 4, 5, 9, 10, 11, 12, 13, 14, 15, 16, 17, 18, 19 and Supplementary Videos S1–S11; the built-in ‘volrenGreen.col’ colour filter was used for Figs. 8, 16b,d–i. Different anatomical parts were independently segmented to obtain the final rendered colorized images seen in Figs. 11, 12, 13, 15, 16b,d, and Supplementary Videos S5–S8 and S9. To observe the actual texture of structures after segmentation, and in desired colours, each structure was subjected to the following arithmetic operation: A*(B > 0), where A represents the whole animal and B the segmented structure.

To reconstruct the spaces inside the filter chamber shown in Fig. 14h,i and Supplementary video S9, a task list was conducted within CTAn software as described previously for reconstruction of the air-filled tracheal system of the coffee berry borer beetle ^82^.

In accordance with the micro-CT results (as seen in the Figures), standard anatomical positions are used to describe structures.

For consistency, and to avoid poor or misleading descriptions of any structure or form as a result of undesired deformation, the structures visualized and described in this study were checked and found to exist and maintain their shape and position in each of the specimens that were scanned and reconstructed.

## Supplementary Information

Supplementary Information.

Supplementary Video S1.

Supplementary Video S2.

Supplementary Video S3.

Supplementary Video S4.

Supplementary Video S5.

Supplementary Video S6.

Supplementary Video S7.

Supplementary Video S8.

Supplementary Video S9.

Supplementary Video S10.

Supplementary Video S11.

Supplementary Video S12.

## Data Availability

The datasets generated and analyzed during the course of the study are available from J.A.-T. upon reasonable request.
