# Publisher Correction: Using micro-computed tomography to reveal the anatomy of adult *Diaphorina citri* Kuwayama (Insecta: Hemiptera, Liviidae) and how it pierces and feeds within a citrus leaf

**DOI:** 10.1038/s41598-021-89262-9

**Published:** 2021-04-29

**Authors:** Javier Alba‑Tercedor, Wayne B. Hunter, Ignacio Alba‑Alejandre

**Affiliations:** 1 Department of Zoology, Faculty of Sciences, University of Granada, Campus de Fuentenueva, Granada, Spain; 2 Agricultural Research Service, U.S. Dept. Agriculture, Fort Pierce, FL USA

Correction to: *Scientific Reports* 10.1038/s41598-020-80404-z, published online 14 January 2021

This Article contained an error in the labelling of a Supplementary Information file.

Supplementary 3D model S12 was incorrectly published as Supplementary Videos S12.

This has now been corrected in the HTML version of the article; the PDF version was correct from the time of publication.

