# Supplementary material for: Using micro-computed tomography to reveal the anatomy of adult Diaphorina citri Kuwayama (Insecta: Hemiptera, Liviidae) and how it pierces and feeds within a citrus leaf
Source: Sci Rep. 2021 Jan 14;11:1358. doi: 10.1038/s41598-020-80404-z (PMC7809155; doi:10.1038/s41598-020-80404-z)
Supplement: Supplementary file 1 — Supplementary Information. [file 41598_2020_80404_MOESM1_ESM.pdf]

For: *Scientific Reports*

SUPPLEMENTARY INFORMATION MATERIAL

Using micro-computed tomography to reveal the anatomy of adult *Diaphorina citri* Kuwayama (Insecta: Hemiptera, Liviidae) and how it pierces and feeds within a citrus leaf

Javier Alba-Tercedor[1]*, Wayne B. Hunter[2] and Ignacio Alba-Alejandre[1]

[1] Department of Zoology, Faculty of Sciences, University of Granada, Campus de Fuentenueva, Granada, Spain.

[2] U.S. Dept. Agriculture, Agricultural Research Service, Fort Pierce, Florida, USA.

*Corresponding authors:

Email: jalba@ugr.es (JAT), ignacioalba@gmail.com (IAA)

<u>Supplementary Videos</u> (mp4):

S1. Animated, volume-rendered images showing the external anatomy and abdominal tracheal tubular system of a female *Diaphorina citri*.

S2. Animated, volume-rendered images of a left-lateral view of the abdomen of an adult female *Diaphorina* citri, with progressive bilateral erosive-slices, from the external surface to the median line, showing several structures and the dorsal vessel (heart) in sagittal view.

S3. Animated, volume-rendered images of an adult male *Diaphorina citri* feeding of a citrus leaf, with detail of the leaf structure, stylets, salivary sheaths and feeding apparatus.

S4. Spinning, animated, volume-rendered images of the feeding apparatus, showing the labium and the internal structures of the cibarium and tentorium of an adult male *Diaphorina citri*.

S5. Spinning, animated, volume-rendered images of an adult male *Diaphorina citri* showing the general position of the nervous system and the digestive (including the salivary glands).

S6. Spinning, animated, volume-rendered images of an adult male *Diaphorina citri* showing the feeding apparatus, the digestive system (including the salivary glands) and the nervous system.

S7. Spinning, animated, volume-rendered images of the anterior body of an adult female *Diaphorina citri* showing the digestive, nervous system, salivary glands, labium, stylets bundle, cibarium, cibarial dilator muscles, cibarial pump and maxillary/mandibular cones.

S8. Spinning, animated, volume-rendered images of the digestive of an adult male *Diaphorina citri*.

S9. Spinning, animated, volume-rendered images of an adult male of *Diaphorina citri* with details of the filter chamber structure and its connections with the esophagus and the outer hindgut.

S10. Spinning, animated, volume-rendered images of an adult male of *Diaphorina citri* with details of the right salivary glands.

S11. Spinning, animated, volume-rendered images of an adult female of *Diaphorina citri* with internal details of the brain and other structures such as the antennal glands and tentorium.

Supplementary 3D model to visualize with mobile devices (vxm):

S12. Adult male of *Diaphorina citri* feeding on the abaxial surface of an orange seedling tree leaf.

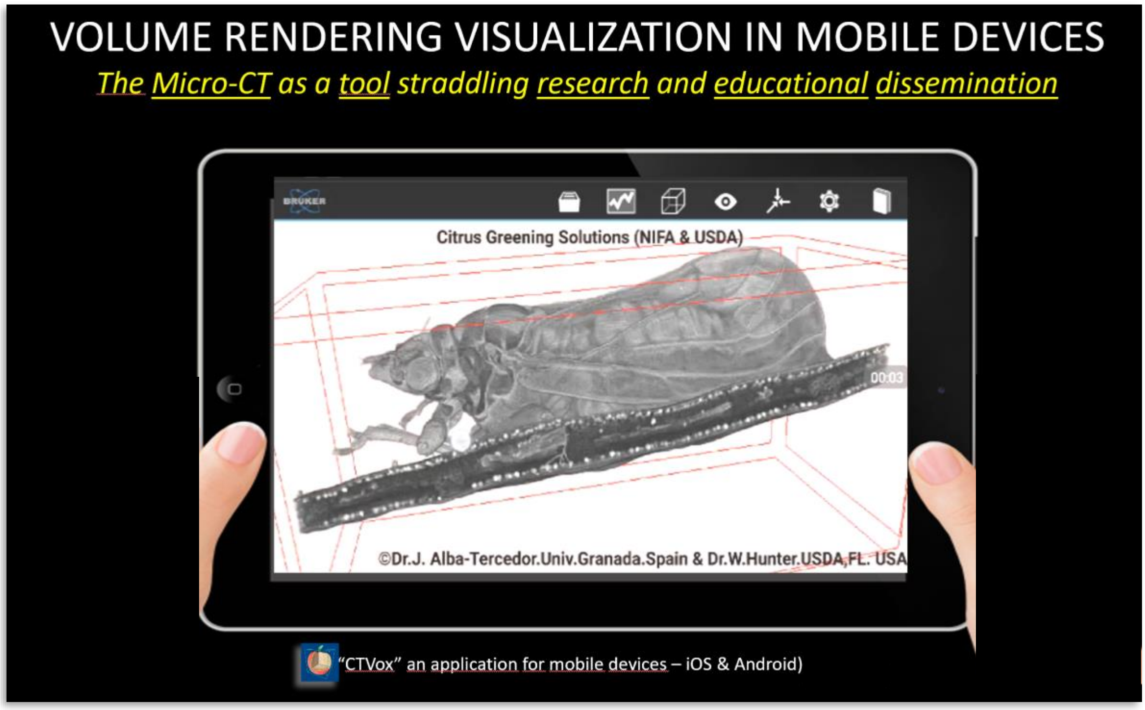

To be visualized with the CTvox app for mobile devices (smartphones and tablets, either with iOS or Android systems). To install CTvox on your device, go through Apple's App Store/Google Play Store, in the usual fashion (the app is free of charge). Instructions can be downloaded in the following links:

1.- For iOS devices:

a) For Ipad:

https://www.bruker.com/fileadmin/user_upload/8-PDF-Docs/Microtomography/CTvoxForIpad.pdf

b) For Iphone:

https://www.bruker.com/fileadmin/user_upload/8-PDF-Docs/Microtomography/CTvoxForIPhone.pdf

2.- For Android devices:

https://www.bruker.com/fileadmin/user_upload/8-PDF-Docs/Microtomography/CTvoxForAndroid.pdf
